# Inhibition of Patched Drug Efflux Increases Vemurafenib Effectiveness against Resistant Braf^V600E^ Melanoma

**DOI:** 10.3390/cancers12061500

**Published:** 2020-06-09

**Authors:** Laurie Signetti, Nelli Elizarov, Méliné Simsir, Agnès Paquet, Dominique Douguet, Fabien Labbal, Delphine Debayle, Audrey Di Giorgio, Valérie Biou, Christophe Girard, Maria Duca, Lionel Bretillon, Corine Bertolotto, Bernard Verrier, Stéphane Azoulay, Isabelle Mus-Veteau

**Affiliations:** 1 Université Côte d’Azur, CNRS, IPMC, 660 Route des Lucioles, 06560 Valobonne, France; lauriesignetti@gmail.com (L.S.); simsir@ipmc.cnrs.fr (M.S.); paquet@ipmc.cnrs.fr (A.P.); douguet@ipmc.cnrs.fr (D.D.); labbal@ipmc.cnrs.fr (F.L.); debayle@ipmc.cnrs.fr (D.D.); 2 Université Côte d’Azur, CNRS, ICN, 28 Avenue Valrose, 06108 Nice, CEDEX 2, France; nelli.elizarov@yahoo.de (N.E.); Audrey.DI-GIORGIO@univ-cotedazur.fr (A.D.G.); Maria.DUCA@univ-cotedazur.fr (M.D.); 3 CNRS, IBPC, Sorbonne Paris Cité, Laboratoire de Biologie Physico-Chimique des Protéines Membranaires, Institut de Biologie Physico-Chimique, University Paris Diderot, 13 rue Pierre et Marie Curie, 75005 Paris, France; valerie.biou@ibpc.fr; 4 Université Côte d’Azur, INSERM, CNRS, C3M, Bâtiment Universitaire ARCHIMED 151 Route Saint Antoine de Ginestière BP 2 3194, 06204 Nice, CEDEX 3, France; christophe.girard@univ-cotedazur.fr (C.G.); Corine.Bertolotto@unice.fr (C.B.); 5 Centre des Sciences du Goût et de l’Alimentation, Université Bourgogne Franche-Comté CNRS, INRA, SSGA, AgroSup Dijon, F-21000 Dijon, France; lionel.bretillon@dijon.inra.fr; 6 Adjuvatis SAS, IBCP, 7 Passage du Vercors—69007 Lyon, France; bernard.verrier@adjuvatis.com

**Keywords:** Patched, melanoma, vemurafenib, chemotherapy resistance: drug efflux, new therapeutic lead

## Abstract

Melanoma patients harboring the BRAF^V600E^ mutation are treated with vemurafenib. Almost all of them ultimately acquire resistance, leading to disease progression. Here, we find that a small molecule from a marine sponge, panicein A hydroquinone (PAH), overcomes resistance of BRAF^V600E^ melanoma cells to vemurafenib, leading to tumor elimination in corresponding human xenograft models in mice. We report the synthesis of PAH and demonstrate that this compound inhibits the drug efflux activity of the Hedgehog receptor, Patched. Our SAR study allowed identifying a key pharmacophore responsible for this activity. We showed that Patched is strongly expressed in metastatic samples from a cohort of melanoma patients and is correlated with decreased overall survival. Patched is a multidrug transporter that uses the proton motive force to efflux drugs. This makes its function specific to cancer cells, thereby avoiding toxicity issues that are commonly observed with inhibitors of ABC multidrug transporters. Our data provide strong evidence that PAH is a highly promising lead for the treatment of vemurafenib resistant BRAF^V600E^ melanoma.

## 1. Introduction

One of the major challenges in the clinical management of cancer is resistance to chemotherapeutics. Multidrug resistance (MDR) has been intensively studied, and overexpression of ATP-binding cassette (ABC) transporters has been considered to be the most prominent underlying mechanism for MDR. Despite research efforts to develop compounds that inhibit the efflux activity of ABC transporters and increase classical chemotherapy efficacy, to date, the Food and Drug Administration has not approved the use of any ABC transporter inhibitor due to toxicity issues [1]. Therefore, it is necessary to find other targets.

The Hedgehog (Hh) signaling pathway controls cell differentiation and proliferation. It plays a crucial role during embryonic development and, in adulthood, it is involved in stem cell homeostasis and tissue regeneration. However, Hh signaling is also involved in cancer development, progression, and metastasis. Aberrant activation of Hh signaling has been observed in many aggressive cancers [2], in particular, in cells exhibiting resistance to chemotherapy such as cancer stem cells or tumor-initiating cells [3]. The Hh receptor, Patched (Ptch1), whose expression is induced upon activation of the Hh pathway, is overexpressed in many cancers, including breast, prostate, ovary, colon, brain, melanoma [4,5,6], and myeloid leukemia [7,8] (see the Human Protein Atlas website http://www.proteinatlas.org/ENSG00000185920-PTCH1/cancer). Studies have even suggested Ptch1 as an early marker of gastric and thyroid cancers [9,10]. We previously showed, for the first time, that Ptch1 has a drug efflux activity and contributes to the resistance of cancer cells to chemotherapy [11]. Remarkably, Ptch1 is not an ABC transporter but uses the proton motive force to efflux drugs. This allows Ptch1 to efflux drugs, at the expense of proton consumption, from the extracellular medium of cancer cells where the extracellular pH is acidic due to the strong glucose consumption (Warburg effect) [12]. This metabolic feature makes Ptch1 drug efflux activity specific to cancer cells. Hence, Ptch1 is a particularly relevant and highly specific therapeutic target for resistant cancers that express Ptch1. This breakthrough allowed us to propose Ptch1 as a new target to enhance the efficiency of classical or targeted chemotherapeutic treatments and decrease the risk of recurrence and metastasis [13]. We then developed a test using Ptch1-overexpressing yeast to identify molecules that were able to inhibit the drug efflux activity of Ptch1 [14]. A first screening of natural compounds purified from marine sponges led to the identification of panicein A hydroquinone (PAH). We showed that this compound strongly inhibited the resistance of Ptch1-overexpressing yeast to doxorubicin (dxr), a chemotherapeutic agent used to treat many cancers, as well as increased the cytotoxic effect of dxr in melanoma cells and strongly inhibited in vitro dxr efflux [15]. The screening of a chemical library allowed us to identify a second inhibitor, methiothepin, which increases the efficacy of dxr against adrenocortical carcinoma cells, in vitro and in vivo [16]. These discoveries suggest that the use of inhibitors of Ptch1 drug efflux activity in combination with classical chemotherapy, such as doxorubicin, could be a novel way to circumvent drug resistance, recurrence and metastasis of tumors expressing Ptch1.

Around 45–50% of cutaneous melanomas have mutations in the BRAF serine/threonine kinase. These patients are treated with vemurafenib. This targeted chemotherapy presents heterogeneous clinical responses, and almost all patients who experience an initial response to vemurafenib ultimately acquire resistance and relapse [17]. In the present study, we have performed the chemical synthesis of the Ptch1 drug efflux inhibitor, PAH, and some analogues, and conducted a preliminary structure activity relationship (SAR) study to enable the identification of a key pharmacophore. We showed that PAH enhances the efficacy of vemurafenib against BRAF^V600E^ melanoma cells, in vitro and in vivo, by directly interacting with Ptch1 and inhibiting vemurafenib efflux. Our results suggest that the use of this inhibitor of Ptch1 drug efflux in combination with vemurafenib could be a promising therapeutic option to improve vemurafenib efficacy against resistant BRAF^V600E^ melanomas.

## 2. Results

### 2.1. Ptch1 Is Expressed in Melanoma and Contributes to the Efflux of Chemotherapy Agents out of Cells

**Figure 1:**
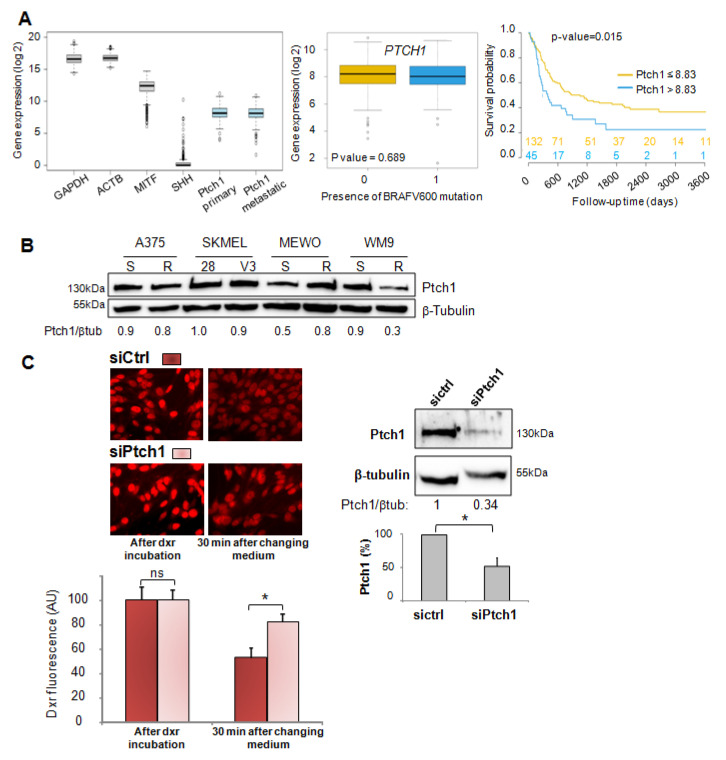
Ptch1 is expressed in patients-derived melanoma specimens and melanoma cell lines, and transports chemotherapy agents out of cells. (**A**). Ptch1 is expressed in patients-derived melanoma specimens and decreases survival in metastatic patients. Left panel: Distribution of gene expression level of *PTCH1*, *GAPDH*, *ACTB*, *MITF*, and *SHH*. Middle panel: Distribution of *PTCH1* gene expression by the presence or the absence of any BRAF^V600^ mutation for metastatic samples (0: no mutation (*n* = 217), 1: presence of a mutation (*n* = 151). Right panel: Kaplan–Meier analysis of overall survival for a subset of patients with metastatic disease and did not receive immunotherapy. Patients were grouped by their level of Ptch1 protein expression (low: Ptch1 expression ≤ 8.83, high: Ptch1 expression > 8.83). The number of patients in each group over time is displayed at the bottom of the graph. (**B**). Ptch1 is expressed in melanoma BRAF^V600^ mutant cell lines. Western blots were performed on extracts from various melanoma cell lines with antibodies directed against Ptch1. (**C**). Ptch1 contributes to the efflux of doxorubicin from melanoma cells. Ptch1 protein expression (right panel) and intracellular doxorubicin (dxr; left panel) were analyzed after transfection of MeWo cells with Ptch1-siRNA or negative-control-siRNA. After incubation with dxr, 3 coverslips were fixed for dxr loading control; the other coverslips were incubated with efflux buffer and fixed. Dxr fluorescence images were acquired using a 40× objective, and dxr fluorescence was quantified using ImageJ software for about 100 cells per condition per experiment. Ptch1 and β-tubulin signals on Western blots were quantified using ImageJ software. All data presented are the mean ± SEM of 3 independent experiments. Significance is attained at *p* < 0.05 (*), ns: no significant difference. The uncropped blots and molecular weight markers of Figure 1B,C are shown in Figure S7.

Normalized gene expression data and matching clinical information for cutaneous melanoma tumors were downloaded from The Cancer Genome Atlas (TCGA), then separated into primary tumor samples (*n* = 103) and metastatic tumor samples (*n* = 368). The distribution of *PTCH1* gene expression level for all TCGA patients compared to genes known to be well expressed in melanoma (GAPDH, ACTB, MITF) indicates that *PTCH1* is well expressed in primary and metastatic samples (Figure 1A left). We did not observe a significant difference in the distribution of *PTCH1* gene expression between tumors carrying or not BRAF^V600^ mutation for primary or metastatic samples (Figure 1A middle).

In this cohort, the Kaplan–Meier analysis for a subset of patients with metastatic disease who did not receive immunotherapy indicated that a high level of Ptch1 in patient samples significantly correlated with a lower overall survival time (Figure 1A right). Patients were grouped according to their level of Ptch1 expression (low: samples with Ptch1 expression ≤ 8.83, high: samples with Ptch1 expression > 8.83). A significant difference in survival was observed (log-rank *p*-value = 0.0146). The same analysis for primary tumor did not show any significant difference.

Western blots were performed on extracts from four melanoma cell lines that are sensitive or resistant to chemotherapy, and strong expression of Ptch1 was found in all of the studied cell lines (Figure 1B). Interestingly, the depletion of Ptch1 using specific silencing RNA in the MeWo melanoma cell line induced cell retention of doxorubicin (dxr), a fluorescent chemotherapeutic drug commonly used to treat many types of cancers, while control cells showed a strong decrease of intracellular dxr fluorescence 30 min after the removal of this drug from the medium (Figure 1C). In this experiment, MeWo cells were seeded on coverslips and transfected with 40 nM Ptch1-siRNA or negative-control-siRNA. Ptch1 protein expression (right panel) and intracellular dxr (left panel) were analyzed 16 h after transfection. After 2 h of incubation with dxr, 3 coverslips were fixed for dxr loading control. The other coverslips (triplicate per condition) were incubated with efflux buffer for 30 min and fixed. Dxr fluorescence was imaged acquired and quantified using ImageJ software for about 100 cells per condition per experiment. Results indicate that Ptch1 contributes to dxr efflux in these cells.

### 2.2. Panicein A Hydroquinone Obtained by Chemical Synthesis Enhances the Sensitivity of Melanoma Cells to Doxorubicin by Inhibiting the Drug Efflux

**Figure 2:**
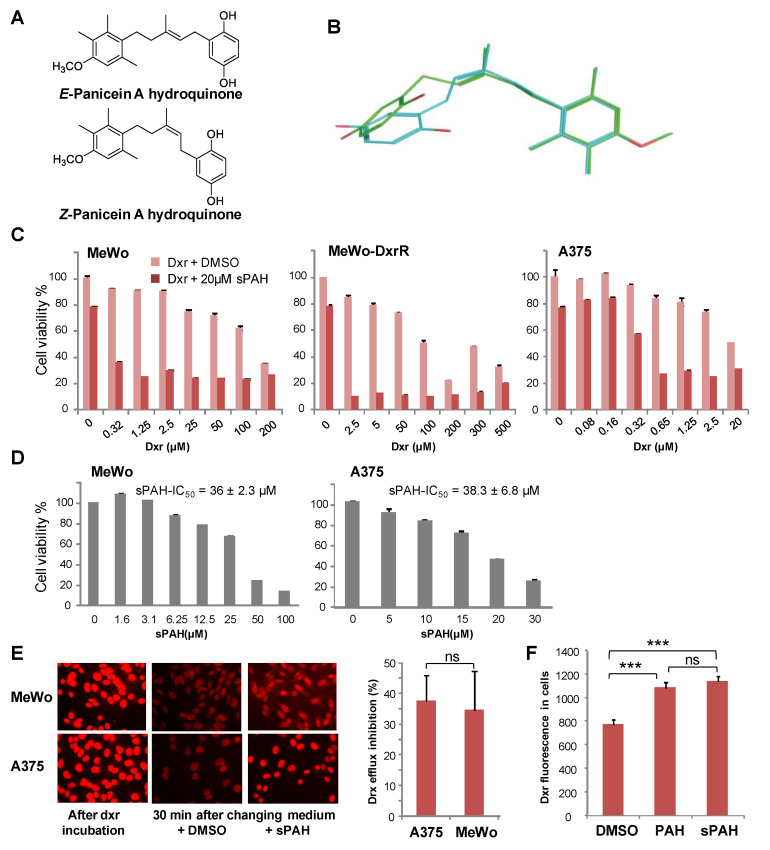
The chemically synthesized PAH is as effective as natural PAH in increasing cytotoxicity and inhibiting the efflux of doxorubicin. (**A**) Stereoisomers of PAH. (**B**) 3D structures of E and Z forms of PAH are very similar. The E form is in green and the Z form is in cyan. (**C**) sPAH increases doxorubicin cytotoxicity in various melanoma cell lines. Cell viability was measured after treatment with increasing concentrations of dxr in the presence of DMSO or 20 µM sPAH on MeWo cells, MeWo cells rendered resistant to dxr (MeWo-DxrR) and A375 cells. (**D**) sPAH cytotoxicity against melanoma cells. Cell viability was measured after treatment with increasing concentrations of sPAH on A375 or MeWo cells. The mean sPAH-IC_50_ values calculated from 3 independent experiments are represented. (**E**) sPAH inhibits the dxr efflux activity of Ptch1. A375 or MeWo cells were seeded on coverslips and incubated with dxr. After 2 h, 3 coverslips were fixed for dxr loading control. The other coverslips were incubated with DMSO or sPAH and fixed. Dxr fluorescence was imaged and quantified using ImageJ software for about 100 cells per condition per experiment. The inhibition of dxr efflux by sPAH for each cell lines is reported. (**F**) Synthetic PAH inhibits the dxr efflux activity of Ptch1 as efficiently as natural PAH. Dxr fluorescence in MeWo cells was quantified after 30 mins in buffer containing DMSO, natural PAH, or synthetic PAH as described in E. All histograms represent the mean ± SEM of 3 independent experiments. Significance is attained at *p* < 0.05 (*) (***: *p* < 0.0005), ns: no significant difference.

Given that natural panicein A hydroquinone (PAH), identified as the first Ptch1 drug efflux inhibitor, is not readily available, the molecule was prepared by chemical synthesis. Starting from previously published work [18], an additional step allowed us to obtain the desired molecule (see Supplementary Materials). The 10-step synthesis, based on a key microwave accelerated Claisen rearrangement, yielded a mixture of E and Z stereoisomers (3:2 ratio) of PAH at 7% total yield (Figure 2A). To produce a sufficient quantity, the synthesis was conducted on a scale of several tens of grams of starting material, demonstrating the robustness of the optimized procedure. As natural PAH is exclusively composed of E isomer, we wanted to separate the E and Z stereoisomers or isomerize the synthesized PAH mixture to the E configuration. Our attempts were unfortunately not successful, and we decided to test the synthetic PAH (sPAH) as a mixture of both stereoisomers.

Melanoma cells from the MeWo cell line and the BRAF^V600E^ mutant cell line A375 were treated with increasing concentrations of dxr, with or without natural or synthetic PAH for either 48 or 24 h, before assessment of cell viability. Results showed that sPAH strongly increased the sensitivity to dxr of cells from both cell lines (Figure 2C, Table 1), and was as effective as the natural PAH (Table 2). Interestingly, our results showed that sPAH also strongly increased drx cytotoxicity in MeWo cells rendered resistant to dxr (MeWo-DxrR) (Figure 2C, Table 1). One can observe that sPAH at 20 µM was slightly cytotoxic by itself. We then treated A375 and MeWo cells with increasing concentrations of sPAH for either 24 or 48 h, and we calculated that the IC_50_ of sPAH in these melanoma cells was of about 40 µM (Figure 2D). This indicates that the effect observed in combination with dxr did not result from additive cytotoxicities of each compound.

Epifluorescence microscopy was used to measure the effect of sPAH on dxr efflux in melanoma cells. As exemplified in Figure 2E, the amount of dxr accumulated in the nuclei of A375 and MeWo cells after incubation with dxr was drastically reduced after 30 min of incubation with an efflux buffer, and the presence of sPAH in the efflux buffer allowed the cells to retain a significant amount of dxr. Quantification of the dxr amounts in cells showed that sPAH inhibited the efflux of dxr from A375 and MeWo cells by 30 to 40%. We demonstrated that synthetic PAH is able to inhibit the efflux of dxr in melanoma cells as efficiently as the natural PAH (Figure 2F). The IC_50_ values of natural and synthetic PAH obtained in the presence of dxr are very similar (Table 2), suggesting the Z stereoisomer is as active as the natural E configurational isomer of PAH. In order to compare the 3D structures of E and Z forms of PAH, 60 and 62 conformers for E-PAH and Z-PAH, respectively, were generated by using chemoinformatic software. The two ensembles of conformers were aligned and compared using the program SENSAAS. The good *gfit* score of 0.761 indicates that the E and Z isomers are almost similar (a *gfit* score ranges from 0 (dissimilar) to 1 (perfect similarity)) (Figure 2B). The comparable activities of the natural E-PAH and the synthetic mixture of E-PAH and Z-PAH can be explained by the flexibility of the pentene linker that allows some E and Z stereoisomers to superimpose very well and to position the two hydroxyls of the benzene ring in such a way that interactions with same residues can be considered (Figure 2B). Therefore, we used the E/Z stereoisomer mixture of the synthetic PAH (sPAH) for further study of the in vitro and in vivo activity of PAH.

### 2.3. The Hydroquinone Moiety Is Essential for PAH Inhibition of Ptch1 Drug Efflux

**Figure 3:**
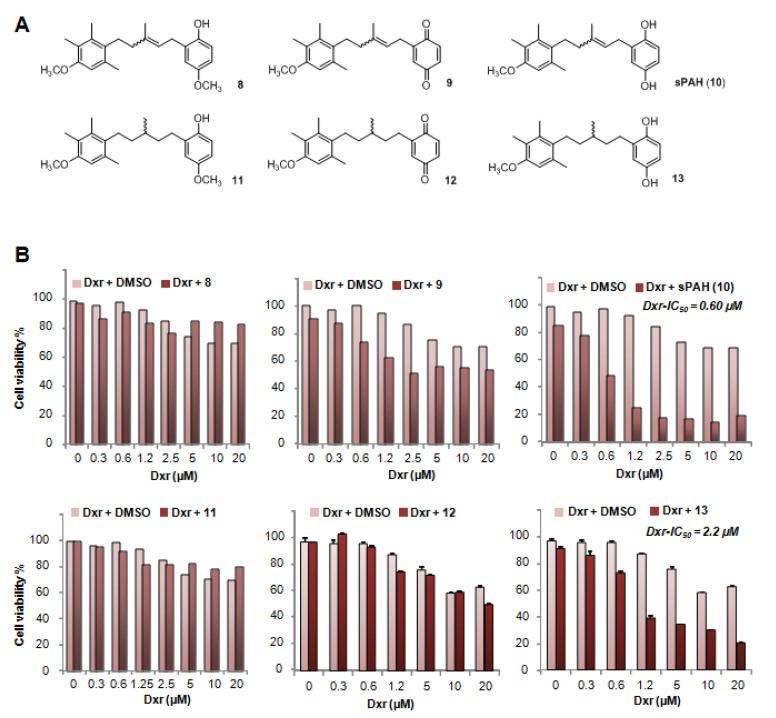
The hydroquinone moiety of PAH is essential to increase doxorubicin efficacy. (**A**) PAH precursors and analogues. (**B**) Effect of PAH precursors and analogues on dxr cytotoxicity. Cell viability was measured after treatment with increasing concentration of dxr in the presence of DMSO or 20 µM sPAH (10), PAH precursors 8 and 9, or PAH analogues 11, 12, and 13 in MeWo cells. Dxr-IC_50_ values calculated in the presence of sPAH or PAH analogue 13 are presented.

In order to identify the key pharmacophores of the PAH, we performed SAR analyses. At this preliminary stage, we tested the activity of 20 µM of two PAH precursors and three synthesized PAH analogues on MeWo and A375 cells (Figure 3A) to focus on the importance of the hydroquinone part and the central double bond. As shown in Figure 3B and Figure S1, the quinone precursor 9 very weakly increased the cytotoxicity of dxr against melanoma cells as compared to PAH, indicating that the quinone form is not able to increase chemotherapy efficacy. Interestingly, precursor 8, where one of the hydroxyl moieties of the hydroquinone is protected by a methyl group, had no effect on dxr cytotoxicity, indicating that this hydroxyl group is crucial. Compound 13 is a hydrogenated form of sPAH where, the central double bond is no longer present. The IC_50_ of dxr for molecules 8, 9, 11, and 12 could not be determined, owing to greater than 50% viability. The IC_50_ of dxr calculated in the presence of 13 is approximately 4-fold higher than that calculated in the presence of PAH in MeWo cells but comparable to that of PAH in A375 cells. These results show that the hydroquinone moiety is essential for sPAH activity as a Ptch1 drug efflux inhibitor, and that the loss of the double bond slightly reduces its activity.

### 2.4. sPAH Increases the Efficacy of Doxorubicin on Melanoma Cells Xenografted in Chick Eggs without Toxic Effects

**Figure 4:**
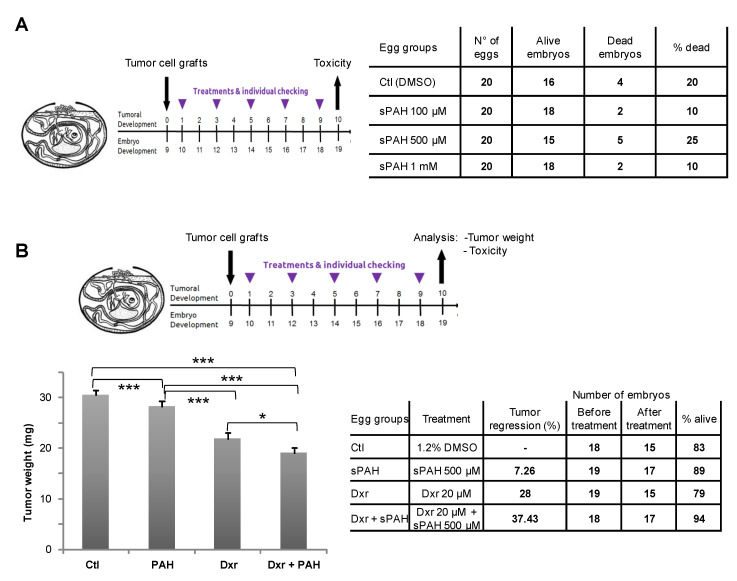
sPAH increases the efficacy of doxorubicin on melanoma cells xenografted in chick eggs without toxic effect. MeWo cells were grafted on the eggs chorioallantoic membrane (CAM). (**A**) Toxicity analysis. Chick embryos were treated with increasing doses of sPAH for 10 days. Then, the dead embryos were counted, and abnormalities were observed on surviving embryos. (**B**) Effect of the association sPAH and dxr on melanoma tumors. One day after inoculation of MeWo cells on the CAM, the tumors were treated by adding 100 µL of vehicle, dxr alone, sPAH alone or with dxr. After 10 days, the tumors were cut away from the CAM tissue and weighed. Significance: *: 0.05 ≥ *p* value > 0.01; ***: 0.001 ≥ *p* value; ns: no significant difference.

MeWo cells were grafted on the chorioallantoic membrane (CAM) after drilling a small hole through the eggshell. Eggs were then randomized in groups of 20 eggs for treatments. Chick embryos were first treated with increasing doses of sPAH alone in order to evaluate sPAH toxicity (Figure 4A)**.** After 10 days of treatment, the dead embryos were counted and abnormalities in 22 checkpoints (head: size, closure, eyes, ear, face, branchial arc derivatives, mobility; body: size, axis deformation, ventral and dorsal closures, caudal formation, sexual area; limbs: size, axis morphology, mobility; skin: appendage formation, attachment, blood vessel; extra-embryonic structures: vascularization, transparency, attachment, blood vessels) were observed in surviving embryos. No acute toxicity was observed even at the highest dose of 1 mM. The percentage of death between 10 and 25% was normal without link with sPAH treatment, and no abnormalities were observed in surviving embryos.

We evaluated the effect of sPAH on dxr treatment of melanoma tumors initiated from MeWo cells (Figure 4B). When tumors became detectable (1 day after inoculation of MeWo cells on the CAM), they were treated by adding 100 µL of vehicle, dxr alone, sPAH alone or in combination with dxr. After 10 days of treatment, the tumors were carefully cut away from normal CAM tissue and weighed. We observed that dxr alone induced a 28% reduction in tumor weight with respect to control, and that association with sPAH enhanced the tumor regression to 37% while sPAH by itself had a very weak effect on tumor development. Mortality rates were normal, and no abnormalities were observed in the surviving embryos.

### 2.5. sPAH Enhances the Sensitivity of Melanoma Cells to Cisplatin

We previously showed that Ptch1 drug efflux inhibition enhanced the cytotoxicity of dxr against adrenocortical carcinoma cells, but also that of cisplatin, another well known chemotherapeutic drug [19], suggesting that both cisplatin and dxr are substrates of Ptch1 [16]. Therefore, we tested the effect of sPAH on the cytotoxicity of cisplatin in the melanoma cell lines MeWo and A375. Interestingly, the IC_50_ of cisplatin was significantly decreased in the presence of sPAH in both MeWo and A375 cells, indicating that sPAH also increases the cytotoxicity of cisplatin against melanoma cells (Figure S2).

### 2.6. sPAH Enhances the Sensitivity of BRAF^V600E^ Melanoma Cells to Vemurafenib

**Figure 5:**
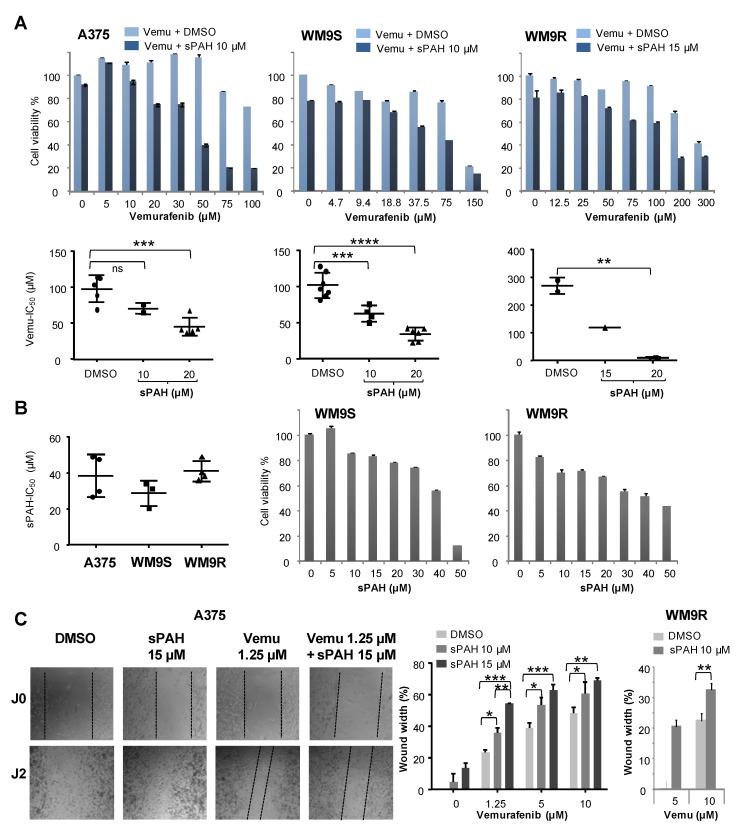
sPAH increases the sensitivity of BRAF^V600E^ melanoma cells to vemurafenib. (**A**) sPAH increases vemurafenib cytotoxicity in three BRAF^V600E^ melanoma cell lines. Cell viability was measured after treatment with increasing concentrations of vemurafenib in the presence of DMSO or sPAH in A375, WM9S, and WM9R cells. Vemurafenib IC_50_ values calculated in the presence of DMSO or at various concentrations of sPAH are reported. (**B**) sPAH cytotoxicity for BRAF^V600E^ melanoma cells. Cell viability was measured after treatment with increasing concentration of sPAH in A375, WM9S, and WM9R cells. sPAH IC_50_ values are reported. (**C**) sPAH increases vemurafenib effect on cell migration. After wounding, A375 or MW9R cells were treated with vemurafenib, sPAH, or vemurafenib + sPAH. Pictures were taken immediately and 48 h after wounding. The width of the wound was measured using ImageJ software and reported as a percentage of final wound width/initial wound width. All data presented are the mean ± SEM of 3 independent experiments. Significance is attained at *p* < 0.05 (*) (**: *p* < 0.005, ***: *p* < 0.0005); ns: no significant difference.

Vemurafenib is a targeted chemotherapy agent which interrupts the BRAF/MEK step in the BRAF/MEK/ERK pathway when BRAF has the V600E mutation [20,21]. In the present study, we used three melanoma cell lines carrying the BRAF^V600E^ mutation: A375, WM9S sensitive to vemurafenib, and WM9R rendered resistant to vemurafenib. Cells from these three cell lines were treated with increasing concentrations of vemurafenib in the presence of DMSO or sPAH for 24 h before assessment of cell viability. Results show that sPAH significantly increased the sensitivity of A375 and WM9S cells to vemurafenib (Figure 5A). Remarkably, sPAH also increased the effectiveness of vemurafenib against WM9 cells rendered resistant to vemurafenib (WM9R) with a decrease by a factor of ten of the IC_50_ of vemurafenib in the presence of sPAH 20 µM (Figure 5A). Note that the concentrations of sPAH used (between 10 and 20 µM) have a slight cytotoxic effect in line with sPAH-IC_50_ calculated of 38.3 ± 6.8, 28.6 ± 5.1, and 41 ± 6.2 µM for A375, WM9S, and WM9R respectively (Figure 5B). Therefore, the cytotoxicity of sPAH itself at the concentration used was not sufficient to explain the strong increase of vemurafenib cytotoxicity observed.

The effect of the combination sPAH and vemurafenib on cell migration was assessed using a wound-healing assay (Figure 5C). Experiments revealed that sPAH also increased vemurafenib efficacy against the A375 cells’ ability to migrate. Indeed, 48 h after wounding, the closing area was significantly greater when sPAH was added to vemurafenib (Figure 5C). Remarkably, the same effect was observed on cells resistant to vemurafenib (WM9R). This indicates that the combination of sPAH and vemurafenib more significantly inhibited the wound-healing and, therefore, cell migration than vemurafenib alone.

### 2.7. sPAH Inhibits Doxorubicin, Vemurafenib and Cholesterol Efflux by Directly Binding to Ptch1

**Figure 6:**
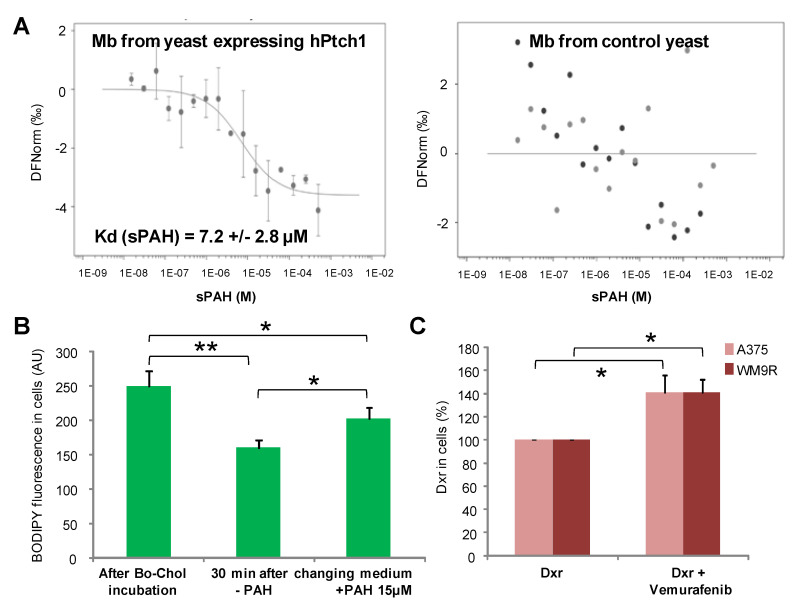
sPAH inhibits doxorubicin, vemurafenib and cholesterol efflux by directly binding to Ptch1. (**A**) Direct binding of sPAH to Ptch1. Membranes from yeast expressing hPtch1 or hSmoothened proteins labeled with tris-NTA-NT647 fluorescent probe were incubated with sPAH and analyzed by microscale thermophoresis (MST). Kd reported is the mean of the Kd values determined in 3 independent experiments. (**B**) sPAH inhibits BODIPY-cholesterol efflux. A375 cells were seeded on coverslips and incubated with BODIPY-cholesterol (Bo-chol) for 2 h. Three coverslips were fixed for Bo-chol loading control, while the others were incubated with DMSO or sPAH and fixed. (**C**) Vemurafenib inhibits doxorubicin efflux. A375 and WM9R cells were seeded on coverslips, incubated with dxr in the absence or the presence of vemurafenib for 1 h and fixed. BODIPY and dxr fluorescence were imaged and quantified using ImageJ software for about 100 cells per condition per experiment. All data presented are the mean ± SEM of 3 independent experiments. Significance is attained at *p* < 0.05 (*) (**: *p* < 0.005).

Using microscale thermophoresis (MST), we showed that sPAH binds to membranes prepared from yeast expressing human Ptch1 with a Kd around 7 µM, while sPAH does not bind to membranes prepared from yeast expressing another human membrane protein, Smoothened (Figure 6A). These results demonstrate that sPAH directly interacts with Ptch1 and that Ptch1 is the target of sPAH.

We previously showed that Ptch1 transports cholesterol [22], and subsequently measured the effect of sPAH on the efflux of BODIPY-cholesterol, a fluorescent derivative of cholesterol, from A375 cells. As shown in Figure 6B, the amount of BODIPY-cholesterol accumulated in cells was drastically reduced after 30 min of incubation with an efflux buffer, and the presence of sPAH in the efflux buffer allowed the retention of a significant amount of BODIPY-cholesterol in cells, suggesting that sPAH inhibited the efflux of cholesterol in A375 cells.

In order to assess if vemurafenib was a substrate of Ptch1, we measured the efflux of dxr from A375 and WM9R cells in the presence of vemurafenib by fluorescence microscopy. Figure 6C reports the mean percentage of dxr accumulated in cells in the presence of vemurafenib relative to dxr accumulated in the absence of vemurafenib. Interestingly, when A375 or WM9R cells were incubated with dxr in the presence of vemurafenib, we observed that the amount of dxr in cells was significantly increased, suggesting that vemurafenib competes with dxr in Ptch1 efflux activity and is a substrate of Ptch1.

**Figure 7:**
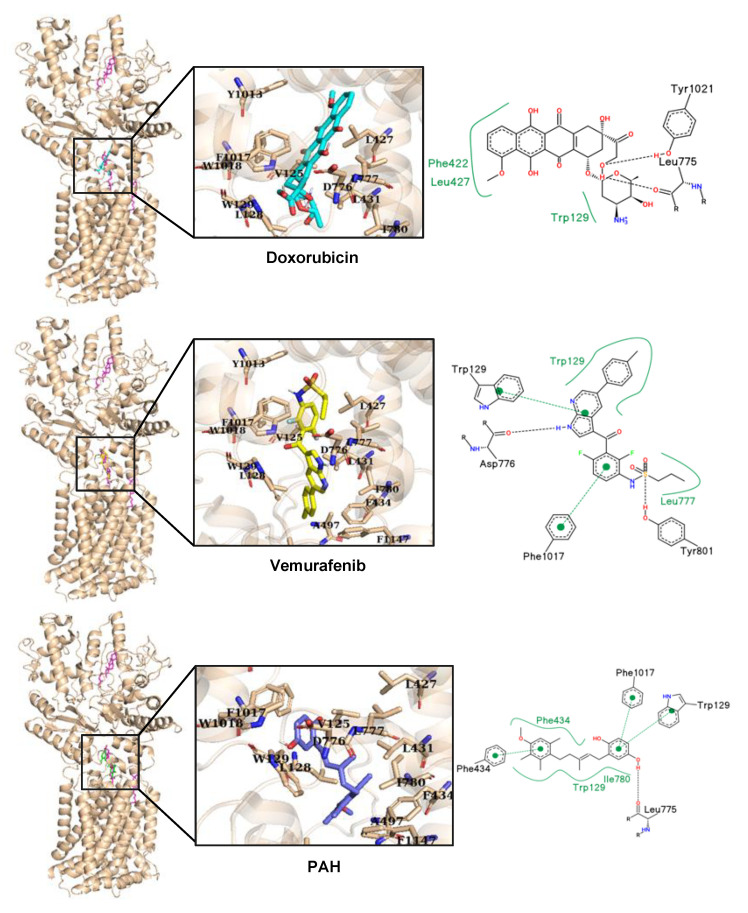
Docking of doxorubicin, vemurafenib, and PAH in the human Ptch1 protein. Chain A of pdb 6n7h with cholesterol (magenta) and molecules: doxorubicin (cyan, top), vemurafenib (yellow, middle), and PAH (blue, bottom) binding in the same binding cavity when docked using Vina. All ligands interact with similar amino acids (see Table 3). Binding interactions between docked molecules and Ptch1 amino acids are represented in black dash for H-bonds and green for hydrophobic interaction/pi-stacking.

As Ptch1 cryo-EM structure has been solved, we performed an in silico docking analysis to visualize the possible binding sites of doxorubicin, vemurafenib, and PAH on Ptch1. Ptch1 structures harbor several cholesterol binding sites as is the case with the structure 6n7h used for in silico docking analyses [23], but one is shown on many structures and is also the site of palmitate binding from Shh. We call it the “central binding cavity”. The docking was performed first on the whole structure, then, given that the poses with the lowest scores were in the central cholesterol binding cavity, docking was subsequently performed by targeting this cavity. The analysis was performed on the 10 best poses of the docking (ranked by score). Because both the amino acids involved in the interaction with the ligands and the nature of interactions were similar among those poses, only one per ligand is presented (Figure 7). Among the amino acids surrounding the cholesterol in the central cavity, five are conserved in proteins from Patched family, of which two have side chains directed toward the cholesterol (Leu427 and Ala497; Figure S3). We also observed that single nucleotide variations in seven of the residues surrounding the cholesterol are responsible for diseases according to the BioMuta database (Table 3). Being built between loops, this cavity is flexible and should be able to accommodate many types of ligands thanks to a large number of aromatic amino acids with polar groups (tyrosines and tryptophans) and some polar residues among the hydrophobic ones. As shown in Figure 7, the best docking poses for dxr, vemurafenib, and PAH are superimposed on cholesterol. We observed at least one hydrogen bond with nearby amino acids for dxr, vemurafenib, and PAH (Leu775 or Asp776), both with the oxygen of the peptide bond. Interestingly, when Asp776 is mutated to glycine, the probability of a damaging phenotype is high (with a score of 0.87 according to the BioMuta database) supporting the importance of this amino acid for Ptch1 function. Another amino acid predicted to interact with dxr, vemurafenib and PAH is Trp129, either by pi-stacking or hydrophobic interaction. This docking analysis revealed that PAH engages more specific interactions with the hydroquinone moiety than with the other half of the molecule which seems to undergo more hydrophobic and less specific interactions. This is in good agreements with the SAR study showing that hydroquinone moiety is very important for the inhibition of Ptch1 drug efflux activity.

### 2.8. sPAH Enhances the In Vivo Effect of Vemurafenib in BRAF^V600E^ Melanoma Cells Xenografted in Mice

Metabolic stability is crucial in drug discovery and development, since it impacts parameters, such as half-life, which are very important in defining the pharmacological and toxicological profile of drugs. Therefore, before testing sPAH in mice, we determined the metabolic stability of sPAH in the presence of mice microsomes and NADPH, and we observed that the half-life of sPAH was only 1 min. LC–MS/MS analyses showed that sPAH is rapidly oxidized in its quinone form (**9**; Figure S4), which is an inactive precursor of sPAH (Figure 3), without further degradation. This indicates that sPAH is a substrate of cytochrome P450.

**Figure 8:**
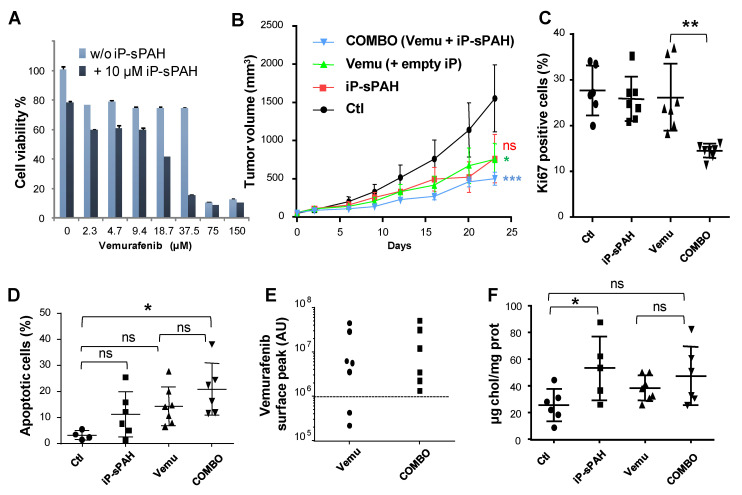
sPAH enhances the effect of vemurafenib on BRAFV600E melanoma cells xenografted in mouse. (**A**) sPAH encapsulated in i-Particles increases the cytotoxicity of vemurafenib. Cell viability was measured after treatment with increasing concentrations of vemurafenib with or without iP-sPAH in A375 cells. (**B**) The addition of iP-sPAH to vemurafenib significantly inhibits the growth of tumors. Mice were injected subcutaneously with A375 cells and treated with either empty i-Particles (empty iP) (Ctl), vemurafenib and empty iP, iP-sPAH, or a combination of vemurafenib and iP-sPAH. Tumor size was measured every 3 to 4 days. At day 23 animals were sacrificed, and tumors were excised and subsequently snap frozen using OCT. (**C**) The addition of iP-sPAH to vemurafenib significantly reduced the number of proliferative cells in tumors. Tumor sections were submitted to Ki67 immunostaining for quantification of proliferative cells using fluorescence microscopy and ImageJ software. (**D**) The addition of iP-sPAH to vemurafenib significantly increases the number of apoptotic cells in tumors. Tumor sections were submitted to the colorimetric DeadEND TUNEL System to determine the number of apoptotic tumor cells in each tumor. (**E**) The addition of iP-sPAH to vemurafenib increases the amount of vemurafenib in tumors. The amount of vemurafenib in each tumor extract was quantified by mass spectrometry. (**F**) iP-sPAH increases the amount of cholesterol in tumors. Sterols were extracted from tumor homogenates and analyzed by GC–MS. All data presented are the mean ± SEM of 3 independent experiments. Significance is attained at *p* < 0.05 (*) (**: *p* < 0.005, ***: *p* < 0.0005); ns: no significant difference.

As sPAH is rapidly oxidized to an inactive compound by liver microsomes, we had to formulate it in order to carry out experiments in mice. We used a biocompatible delivery system, i-Particles (iP), developed by Adjuvatis (Lyon, France) to provide a highly concentrated aqueous and injectable solution of sPAH and protect it from in vivo oxidation. These fully metabolizable i-Particles are made of poly(lactic acid). The efficiency of encapsulation of sPAH in i-Particles was high (≥79%), and sPAH-loaded i-Particles produced were extremely reproducible in terms of size and size homogeneity, with an average diameter of 185 nm as characterized by DLS. iP-sPAH exhibited low polydispersity and negative zeta potential values whatever the drug loading (from 1 to 9%) (Figure S5A), and a colloidal stability at +4 °C for at least six months (Figure S5B). A formulation of 2.44 mg of encapsulated sPAH/mL (7 mM) was tested on melanoma cells and results show that sPAH encapsulated in i-Particles (iP-sPAH) was able to increase the cytotoxicity of vemurafenib against A375 cells in a manner that was comparable to the free sPAH (Figure 8A). As expected, sPAH was not metabolized by mice microsomes after 60 min indicating that the sPAH in i-Particles is metabolically stable. Moreover, no behavioral disorders related to product toxicity were observed for 3 days after intraperitoneal administration of this preparation at doses of 1.33, 4, 13.3, and 40 mg/kg (10 mL/kg) in mice.

Therefore, we tested the effect of iP-sPAH in association with vemurafenib in athymic mice xenografted with BRAF^V600E^ A375 melanoma cells. When tumors became detectable, the mice were randomized in 4 groups and treated intraperitoneally with 4 therapeutic cycles which consisted of either vemurafenib 8 mg/kg body weight (5 µL/g in olive oil) 5 days a week, iP-sPAH 5 mg/kg body weight (4 µL/g) every other day, or a combination of vemurafenib and iP-sPAH. Mice from the control group were injected with olive oil (5 µL/g) 5 days a week plus empty i-Particles 4 µL/g every other day. Animals were monitored daily for symptoms of disease and tumor size was measured every 3 to 4 days. At day 23 after the start of treatment, when the first tumors reached a longest tumor diameter of 1.5 cm, the study was terminated, and animals sacrificed. Tumors were excised and subsequently snap frozen using OCT. Although the dose of vemurafenib chosen causes a slightly too strong effect, experience showed that the combination with i-Particles containing sPAH reduced tumor size more significantly (Figure 8B). Remarkably, no obvious signs of undesirable side effects such as weight loss or abnormal behavior of the animals were observed.

Analyses showed that tumors excised from animals treated with the iP-sPAH + vemurafenib combination contained significantly fewer proliferative cells (Figure 8C) and more apoptotic cells (Figure 8D) than tumors excised from animals treated with vemurafenib alone, indicating that sPAH increased the anti-proliferative and anti-apoptotic effects of vemurafenib.

Moreover, quantification of the vemurafenib contained within the tumors showed that extracts from all the tumors treated with the combination iP-sPAH + vemurafenib exhibited a vemurafenib peak area that was greater than 10^6^ units, corresponding to approximately 100 nM, while extracts from two of seven tumors treated with vemurafenib alone showed a vemurafenib peak area of 2.10^5^ to 4.10^5^ units corresponding to the background signal (Figure 8E).

Furthermore, sterols were extracted from tumor homogenates and analyzed by gas chromatography and mass spectrometry (GC–MS). Interestingly, this analysis revealed that tumors treated with iP-sPAH contained significantly more cholesterol than the other tumors (Figure 8F). This increase in cholesterol suggests that sPAH also inhibited cholesterol efflux in tumors in good agreements with in vitro observations.

Vemurafenib is known to selectively block the RAF/MEK/ERK pathway in *BRAF* mutant cells [24,25,26]. Since the BRAF/MEK/ERK pathway is linear, it is possible to relate the BRAF activity to the levels of phosphorylated ERK (pERK) in a Western blot assay. Therefore, we studied the levels of pERK and ERK in extracts from tumors excised from animals by Western blotting (Figure S6) and, in line with previous reports, we observed that vemurafenib treatment reduced ERK phosphorylation. Although the dose of vemurafenib applied causes a slightly too strong effect, this experiment shows that the addition of iP-sPAH to the vemurafenib treatment reduced ERK phosphorylation more significantly than vemurafenib alone. This result can be explained by the increased amount of vemurafenib in tumors, shown in Figure 8E, and is consistent with the increased number of apoptotic cells observed when iP-sPAH was added to the vemurafenib treatment (Figure 8D).

## 3. Discussion

Cutaneous melanoma is a complex disorder characterized by elevated heterogeneity. It is one of the most aggressive types of cancer and one of the leading causes of cancer-related mortality due its metastatic power. Its therapeutic management is a real challenge as it is amongst the solid malignancies most refractory to conventional cancer therapies [27]. Up to 90% of melanomas exhibit aberrant MAPK pathway activation that induces cell cycle deregulation and apoptosis inhibition, and marked improvements in cutaneous melanoma treatment have been achieved by targeting the MAPK signaling pathway. Improved overall survival outcomes were observed with targeted therapies in patients with *BRAF*^V600^ mutant unresectable stage III or stage IV melanoma. Nearly half of patients with metastatic melanomas harbor a valine–glutamine substitution in codon 600 of the serine/threonine kinase BRAF [28]. Vemurafenib, dabrafenib, and encorafenib are BRAF inhibitors (BRAFi) approved by the US Food and Drug Administration (FDA) to treat patients with *BRAF*^V600E^ mutated metastatic melanomas [29]. BRAFi have relatively high response rates; however, patients almost invariably develop disease progression after about 5 months. The addition of a MEK inhibitor to BRAFi extends the median duration of response from 5.6 months to 9.5 months [30,31]. However, some patients develop resistance to BRAF (±MEK) inhibitors [31,32]. Both intrinsic and acquired resistances can be driven by genetic and epigenetic alterations that drive gene expression changes and intratumor heterogeneity which, in turn, enable tumor regrowth and disease relapse [33]. Although significant progress has been made in therapeutic approaches, cutaneous melanoma still represents a major problem worldwide due to its high incidence and the lack of a curative treatment for advanced stages. The discovery of therapeutic compounds for treating advanced melanomas that are resistant to existing therapies is paramount to further improve patient outcomes.

Amongst the mechanisms used by cancer cells to become resistant to treatment, multidrug resistance (MDR) has been intensively studied [34]. A key mechanism underlying MDR is overexpression of ATP-binding cassette (ABC) transporters [35]. However, we recently discovered that the Hedgehog receptor Ptch1, which is overexpressed in many cancers, also pumps chemotherapeutic agents such as doxorubicin out of cancer cell lines that were derived from melanoma and adrenocortical carcinoma (ACC), thereby conferring resistance to chemotherapy [11,16].

In the present study, we report that Ptch1 is strongly expressed in primary and metastatic specimens from a cohort of 471 cutaneous melanoma patients from The Cancer Genome Atlas (TCGA), and that a high expression level of Ptch1 in patient-derived metastatic samples significantly correlated with a lower overall survival time (Figure 1A). Ptch1 is endogenously expressed in various melanoma cell lines, and we found that decreased Ptch1 expression strongly inhibited the efflux of doxorubicin, indicating that Ptch1 is involved in doxorubicin efflux in melanoma cells with or without the BRAF mutation (Figure 1C). Interestingly, we observed that the presence of the BRAF^V600E^ inhibitor, vemurafenib, strongly inhibited the accumulation of doxorubicin in melanoma cells, and in silico docking studies suggested that doxorubicin and vemurafenib bind to Ptch1 at the cholesterol binding site (Figure 7). These observations suggest that vemurafenib could also be transported by Ptch1 and that Ptch1 could contribute to melanoma cell resistance to vemurafenib, and also that the drugs are exported through the same mechanism as cholesterol.

In a previous study, we identified panicein A hydroquinone (PAH) as an inhibitor of the doxorubicin efflux activity of Ptch1 [15]. Due to the limited availability of PAH that is naturally produced by marine sponges, production of synthetic PAH was necessary. To the best of our knowledge, PAH synthesis has not previously been reported; thus, we carried out the first chemical synthesis of PAH in order to further characterize its activity in vitro and in vivo. While natural PAH is exclusively observed in the E configuration, the chemical synthesis led to a mixture of E and Z stereoisomers that could not be separated. However, the mixture of both stereoisomers proved to be as effective as the natural compound in increasing the cytotoxicity of doxorubicin and inhibiting its efflux in melanoma cells (Figure 2 and Table 2). This suggests that the configuration of the double bond does not have a strong influence on the activity. As revealed by the energy minimization study, this may be due to its sufficiently flexible backbone that allows both stereoisomers to superimpose for a large part of the structure of the molecule, coming into proximity with the functional group in such a way that the same interactions can be considered. However, the hydrogenated form of PAH (13) showed a significant loss of activity, indicating that too much flexibility is detrimental. More interestingly, the absence of compound 8 activity highlights the importance of the hydroxyl function of the hydroquinone. This data is perfectly in accordance with the docking study where this function is engaged in a hydrogen bond with Leu775, which could be a key interaction for the activity. This is also confirmed by the fact that the quinone form of PAH (9) is not active, maybe due to the inability to interact as a hydrogen bond donor.

Experiments carried out in embryonated eggs have shown that this synthetic PAH mixture (sPAH) is not toxic to the chicken embryos and, when added in combination with doxorubicin, can inhibit melanoma growth more effectively than doxorubicin alone (Figure 4), which is a very encouraging in vivo proof of concept.

We also observed that sPAH was able to increase the cytotoxicity of cisplatin, another chemotherapeutic agent that we previously identified as a substrate of Ptch1 [16], against melanoma cells in vitro (Figure S2). We then wanted to know if sPAH could also enhance the efficiency of targeted chemotherapy such as vemurafenib against BRAF^V600E^ melanoma cells, and found that sPAH strongly increased the cytotoxicity of vemurafenib, even in resistant BRAF^V600E^ melanoma cells (Figure 5). We observed that sPAH itself is slightly cytotoxic for melanoma cells; however, the concentration of sPAH used was not sufficient to explain the strong increase of vemurafenib cytotoxicity induced by sPAH, implying that the combination vemurafenib/sPAH is synergistic, as is the case for combinations of sPAH with doxorubicin or cisplatin. Moreover, wound-healing assays showed that addition of sPAH to vemurafenib significantly reduced the reclosure of wounds compared to vemurafenib alone (Figure 5), suggesting that a sPAH/vemurafenib combination could be more effective against the migration of BRAF^V600E^ melanoma cells than vemurafenib alone.

A microscale thermophoresis study allowed us to demonstrate the direct binding of sPAH to Ptch1, and in silico docking of PAH with the Ptch1 structure revealed that the best poses of PAH are located in the cholesterol central binding cavity, where vemurafenib and doxorubicin also present the best docking score (Figure 6 and Figure 7).

Altogether, our in vitro results suggest that sPAH increases doxorubicin and vemurafenib efficacy by binding to the same pocket as these chemotherapeutic agents on Ptch1 and inhibiting their efflux by Ptch1. The fact that sPAH also inhibited cholesterol efflux strengthens this interpretation.

Before testing sPAH in mice, we studied its metabolic stability and found that sPAH is very rapidly oxidized on contact with mice liver microsomes. The resulting quinone derivative is unfortunately inactive (Figure 3, Figures S1 and S4). Therefore, we decided to encapsulate sPAH in particles made of poly(lactic acid) (iP-sPAH). This delivery system is highly effective in protecting sPAH from metabolic degradation and iP-sPAH proved to be as active as free sPAH in increasing the cytotoxicity of vemurafenib in vitro (Figure 8A). Moreover, experiments performed on mice have shown that iP-sPAH is well tolerated up to high doses by these animals. Based on these results, we subsequently tested the effect of the iP-sPAH/vemurafenib combination on melanoma xenografts in mice. We injected immune-compromised mice with BRAF^V600E^ A375 melanoma, which is a cell line typically used as a model of xenograft melanoma for testing novel anti-melanoma compounds [36,37]. Experiments performed on these mice showed that the addition of iP-sPAH to the vemurafenib treatment inhibited tumor growth more significantly than vemurafenib alone (Figure 8B). This was accompanied by a decrease in proliferation and an increase in apoptosis of tumor cells, indicating that the treatment is more cytotoxic against melanoma cells, which is supported by the in vitro results. In melanoma cells, BRAF^V600E^ causes constitutive activation of the BRAF tyrosine kinase, which phosphorylates and activates the MEK1/2 dual kinase, which in turn phosphorylates and activates its only target, the receptor tyrosine kinase ERK1/2. Vemurafenib inhibits BRAF activation and ERK1/2 phosphorylation [20,24,26]. Analyses of tumor extracts revealed that the addition of iP-sPAH to vemurafenib treatment more significantly inhibited the phosphorylation of ERK1/2 than vemurafenib alone, indicating an increase in the effectiveness of vemurafenib. This effect was due to the increased concentration of vemurafenib in tumors (Figure 8E). Indeed, in the absence of iP-sPAH, we found that some tumors contained little vemurafenib, suggesting that these tumors contained cells that were able to efflux vemurafenib. In the presence of iP-sPAH, all tumors accumulated sufficient vemurafenib to inhibit BRAF and to induce cell apoptosis. All these results strongly suggest that iP-sPAH inhibited the efflux of vemurafenib in melanoma xenografts. Notably, these effects were achieved without obvious undesirable side effects for mice.

We noticed that iP-sPAH alone induced an insignificant decrease in tumor growth (Figure 8B). Given that sPAH inhibited the cholesterol efflux activity of Ptch1 on A375 cells in vitro (Figure 6), we wondered if this effect was not due to an increase of cholesterol in melanoma cells. We therefore quantified the amount of cholesterol in tumor extracts and, indeed, found that tumors treated with iP-sPAH contained significantly more cholesterol than other tumors (Figure 8F), indicating that sPAH inhibited cholesterol efflux mediated by Ptch1 in this system. Previous studies have shown that cholesterol synthesis increases in cancer cells, which helps cancer cell proliferation [38]. However, Lim and co-workers reported, in 2014, that addition of cholesterol to culture medium led to markedly reduced viability of stomach cancer cells [39]. Indeed, in healthy cells, accumulation of free cholesterol has been shown to induce many mechanisms of cellular toxicity, including disrupted function of the integral membrane proteins and signaling proteins that reside in membrane domains, intracellular cholesterol crystallization, oxysterol formation, and the triggering of apoptotic signaling pathways [40]. Thus, we cannot exclude that the increase in cholesterol accumulation caused by the inhibition of Ptch1 cholesterol efflux by sPAH also contributes to toxicity in melanoma cells. In such a hypothesis, the inhibition of Ptch1 efflux activity by sPAH would have dual benefits: to keep the intracellular antineoplastic concentration high and to specifically increase the intracellular cholesterol concentration in cancer cells.

Our data provide strong evidence that PAH is a highly promising lead for the treatment of vemurafenib resistant BRAF^V600E^ melanoma. However, one of the key pharmacophores of sPAH is not stable under biological conditions in living animals and, therefore, in human, since the hydroquinone is metabolized in the inactive quinone form. As the rest of the scaffold is stable and no other modifications have been observed in metabolic experiments, we first plan to replace the hydroquinone part by another group, mainly aromatic, with substitutes that maintain the hydrogen bond interactions. In parallel, an in-depth SAR study will be conducted on other parts of PAH, in particular, on the methoxytrimethylphenyl part, to optimize the activity.

## 4. Materials and Methods

### 4.1. Chemical and Biological Material

Reagents and solvents were purchased from Merck and Carlo Erba Reagents and used without further purification. All reactions involving air- or moisture-sensitive reagents or intermediates were performed under an argon atmosphere. Flash column chromatography was carried out on silica gel columns (Interchim Puriflash silica HP 15 μm) on a Puriflash XS420 system (Interchim). Analytical thin-layer chromatography (TLC) was conducted on Sigma Aldrich precoated silica gel and compounds were visualized by irradiation (254 nm) and/or by staining with ninhydrin and phosphomolybdic acid. 1H and 13C NMR spectra were recorded on a Bruker AC 200 MHz or a Bruker AC 400 MHz spectrometer. Chemical shifts are reported in parts per million (ppm, δ) referenced to the residual 1H resonance of the solvent (CDCl3, δ 7.26; CD3OD δ 3.31; DMSO-*d*_6_ δ 2.50). Doxorubicin hydrochloride and vemurafenib were purchased from Sigma-Aldrich and Selleckchem, respectively. BODIPY-cholesterol was purchased from Avanti (Topfluor, Avanti). Empty i-Particles^®^ made of poly(d,l-lactic acid) only were purchased from Adjuvatis (Lyon, France).

Human melanoma cell lines A375 and MeWo were purchased from ATCC, and cultured in DMEM medium supplemented with 10% fetal bovine serum and penicillin/streptomycin (Invitrogen) at 37 °C in a 5% CO_2_/95% water-saturated air atmosphere. Melanoma cell lines SKMEL 28, SKMEL V3, WM9S, and WM9R were provided by Robert Ballotti (C3M, Nice, France). MeWo-dxrR cells were obtained by adding increasing concentrations of doxorubicin, up to 0.2 µM, in the culture medium over 6 months. These cells were grown in medium supplemented with 0.2 µM dxr. WM9R cells were obtained by adding increasing concentrations of vemurafenib, up to 5 µM, in the culture medium over 6 months. These cells were grown in medium supplemented with 5 µM vemurafenib. HEK cells were grown in 75 mm tissue culture dishes (Falcon, Franklin Lakes, NJ, USA) in Dulbecco’s modified Eagle’s medium (Gibco, Life Technologies, Saint Aubin, France) supplemented with 10% fetal calf serum (Hyclone, Thermo Fisher Scientific GMBH, Ulm, Germany) and 1% penicillin/streptomycin (Gibco, Life Technologies, Saint Aubin, France) in a humidified incubator at 37 °C (5% CO_2_).

### 4.2. Chemical Synthesis of Panicein A Hydroquinone and Analogues

The first steps of the synthesis were conducted as reported [18] with minor modifications, as described in Supplementary Material.

Panicein A hydroquinone (**10**): A solvent mixture of acetone and water in a ratio of 1:1 was prepared. Panicein A (20 mg) was dissolved in 0.3 mL solvent. A suspension of Na_2_S_2_O_5_ (3 eq) in water (0.1 mL) was added dropwise to the starting material. The reaction was stirred at room temperature for 6 h max (see TLC). An extraction with ethyl acetate followed. The organic fractions were washed with saturated NaCl (aq.) solution, dried over Na_2_SO_4_, and evaporated. The crude residue was purified by flash chromatography on silica gel using a gradient of PE/EA (5:1) as the eluent to give 10 as a white solid. Yield = 86%. Rf = 0.105 (Cy/EA 5:1). 1H NMR (400 MHz, CDCl_3_) δ 6.68 (m, 1H), 6.62–6.47 (m, 3H), 5.34 (m, 1H), 3.80 (s, 3H), 3.34 (d, *J* = 7.1 Hz) and 3.25 (d, *J* = 7.1 Hz) (1H, E and Z), 2.78–2.66 (m, 2H), 2.35 (s) and 2.32 (s) (3H, E and Z), 2.26 (s) and 2.22 (s) (3H E and Z), 2.20–2.15 (m, 2H), 2.19 (s) and 2.15 (s) (3H, E and Z), 1.81 (s) and 1.78 (s) (3H, E and Z).13C NMR (101 MHz, CDCl3) δ 155.5, 155.4, 149.6, 149.5, 148.2, 148.1, 138.6, 138.2, 136.1, 136.1, 133.9, 133.8, 131.0, 131.0, 128.4, 123.1, 123.0, 122.6, 121.6, 116.7, 116.7, 116.6, 116.5, 113.9, 113.8, 110.7, 110.6, 55.8, 55.8, 39.7, 32.2, 31.0, 29.5, 29.3, 28.9, 28.2, 23.7, 20.6, 20.5, 16.6, 15.9, 15.9, 12.1. ESI: *m/z* 341.4 (M + H)^+^ (theoretical 341.2).

Compound **11**: Compound **8** was dissolved in ethyl acetate (10 mL solvent for 1 mmol of starting material). Around 1–2 mol% Pd/C (10 mol%) was added, and the reaction mixture was stirred overnight under hydrogen atmosphere. The crude residue was purified by flash chromatography on silica gel using a gradient of PE/EA (5:1) as the eluent to give **11** as a white solid. Yield = 96%. Rf = 0.36 (Cy/EA 5:1). 1H NMR (400 MHz, CDCl3) δ 6.65–6.46 (m, 4H), 3.70 (s, 3H), 3.67 (s, 3H), 2.68–2.36 (m, 4H), 2.23 (s, 3H), 2.14 (s, 3H), 2.06 (s, 3H), 1.70–1.49 (m, 2H), 1.49–1.35 (m, 2H), 1.22 (m, 1H), 0.99 (d, *J* = 6.4 Hz, 3H). 13C NMR (101 MHz, CDCl_3_) δ 155.3, 153.9, 147.6, 135.9, 133.5, 131.9, 130.3, 122.9, 116.0, 115.9, 111.7, 110.5, 55.9, 55.7, 37.1, 36.8, 33.7, 28.1, 27.3, 20.5, 19.7, 15.8, 12.1. ESI: *m*/*z* 357.1 (M + H)^+^ (theoretical 357.2).

Compound **12**: A solvent mixture of acetonitrile and water in ration of 2:1 was prepared. Around 100 mg of **11** was dissolved in 2 mL of solvent and cooled to 0 °C. Under stirring, CAN (2.2 eq) dissolved in 4 mL solvent was added dropwise to the mixture. The mixture was stirred for maximum 4h. The reaction was extracted with ethyl acetate and the crude residue was purified by flash chromatography on silica gel using a gradient of PE/EA (15:1) as the eluent to give **12** as an orange solid. Yield = 79%. 1H NMR (400 MHz, CDCl_3_) δ 6.80–6.68 (m, 2H), 6.60–6.53 (m, 2H), 3.79 (s, 3H), 2.76–2.38 (m, 4H), 2.31 (s, 3H), 2.22 (s, 3H), 2.15 (s, 3H), 1.66–1.54 (m, 2H), 1.53–1.37 (m, 3H), 1.07 (d, *J* = 6.3 Hz, 3H, 9-H). ESI: *m*/*z* 341.5 (M + H)^+^ (theoretical 341.2).

Compound **13**: Compound 10 was dissolved in ethyl acetate (for 1 mmol of starting material 10 mL solvent). Around 1–2 mol% Pd/C (10 mol%) was added, and the reaction mixture was stirred overnight under hydrogen atmosphere. The crude residue was purified by flash chromatography on silica gel using a gradient of PE/EA (5:1) as the eluent to give **13** as light yellow oil. Yield = 97%. 1H NMR (200 MHz, CDCl3) δ 6.61–6.36 (m, 4H), 3.69 (s, 3H), 2.65–2.45 (m, 4H), 2.23 (s, 3H), 2.14 (s, 3H), 2.07 (s, 3H), 1.76–1.07 (m, 5H), 0.99 (d, *J* = 6.0 Hz, 3H). 13C NMR (50 MHz, CDCl3) δ 155.3, 149.5, 147.5, 135.9, 133.6, 131.9, 130.4, 122.9, 116.9, 116.2, 113.4, 110.5, 55.8, 37.0, 36.8, 33.6, 27.8, 27.2, 20.5, 19.7, 15.9, 12.1. ESI: *m*/*z* 343.6 (M + H)^+^ (theoretical 343.2).

### 4.3. Alignment of E and Z Stereoisomers of PAH

The RDKit Open-Source Cheminformatics Software [41] was used to generate up to 100 conformers for each molecule by using the e-LEA3D web server (https://chemoinfo.ipmc.cnrs.fr). This resulted in 60 and 62 conformers for PAH E and Z, respectively. The shape-based alignment program SENSAAS (https://arxiv.org/abs/1908.11267) was used to align and compare the two ensembles of conformers. The alignment with the best shape score (gfit = 0.761) is displayed in Figure 2B. A *gfit* score ranges from 0 (dissimilar) to 1 (perfect similarity).

### 4.4. TCGA Data Analysis

Normalized gene expression data and matching clinical information for cutaneous melanoma (SKCM) tumors were downloaded from TCGA using the R package curatedTCGAData, then separated into primary tumor samples (*n* = 103) and metastatic tumor samples (*n* = 368). Gene expression data was transformed into log2 scale prior to analysis. A count of 1 was added to all expression values to avoid zeros prior to transformation. BRAF^V600^ mutation status was downloaded using the R package GenomicDataCommons (MuTect2 Variant Aggregation and Masking workflow). Survival data was further processed as described in [42]. Statistical significance of the differences in gene expression distribution was performed using the Wilcoxon rank sum test. A Kaplan–Meier analysis was performed using the R package rms (Harrel). Optimal cut-offs were determined using positional scanning [43].

### 4.5. Ptch1 Knock-Down

MeWo cells were transfected with 400 pmol of human Ptch1 Silencer^®^ Select pre-designed siRNA (Ambion, #4392420, s11441 (sense: 5′GCACUUACUUUACGACCUAtt3′; as: 5′UAGGUCGUAAAGUAAGUGCtg3′) or control (medium GC) siRNA oligos (Invitrogen) using Lipofectamine RNAiMAX reagent (Invitrogen) following the manufacturer’s protocol, then seeded in 24-well plates and incubated at 37 °C and 5% CO_2_ for 16 h before Western blotting and dxr efflux measurements.

### 4.6. Cytotoxicity Assays

Cells were seeded in 96-well plates in triplicate and grown in medium to achieve 70 to 80% confluence. Medium was then removed and replaced with 100 µL/well of complete medium containing PAH or DMSO as a control. After 2 h, 100 µL of complete medium containing serial dilutions of dxr, cisplatin or vemurafenib were added. Plates were incubated at 37 °C and 5% CO_2_. After 24 or 48 h, cells were incubated for 3 h at 37 °C with 100 µL/well neutral red (NR) solution (50 µg/mL in medium) following the manufacturer’s protocol. Measurements were made in microplate readers (Multiskan Go Microplate Spectrophotometer from Thermo Scientific). IC_50_ was defined as the concentration that resulted in a 50% decrease in the number of live cells, and IC_50_ values were calculated using GraphPad Prism 6 software.

### 4.7. Wound-Healing Assay

Once cells were confluent in 24-well plates, a wound was created using a p200 tip. Two pictures were taken at two different points of each well immediately after wounding, and 48 h after wounding. Images were taken with Leica DM IRB (5×). The width of the wound was measured using ImageJ software and reported as percentage final wound width/initial wound width.

### 4.8. Efflux Measurements

Dxr efflux measurements were carried out as previously described [16]. Cells were seeded on coverslips in 24-well plates and allowed to grow to 80% confluence. Coverslips were incubated at 37 °C and 5% CO_2_ with 10 μM dxr in physiological buffer (140 mM NaCl, 5 mM KCl, 1 mM CaCl_2_, 1 mM MgSO_4_, 5 mM glucose, 20 mM HEPES, pH 7.4). After 2 h, three coverslips were immediately fixed with 4% PFA for the dxr loading control, rapidly washed with PBS and mounted in SlowFade Gold antifade reagent with DAPI (Invitrogen). The other coverslips (triplicated per condition) were incubated with physiological buffer supplemented with DMSO or 10 µM of PAH under gentle shaking at room temperature and protected from light. After 30 min, coverslips were fixed with 4% PFA, washed, and mounted as described above. For competition on dxr loading, A375 and WM9R cells seeded on coverslips were incubated for 1 h at 37 °C and 5% CO_2_ with 10 μM dxr in physiological buffer in the presence or the absence of 100 µM vemurafenib. Images were acquired with a Zeiss Axioplan 2 fluorescence microscope coupled to a digital charge-coupled device camera using a 40×/1.3 Plan NeoFluar objective and filters for Alexa 594. Dxr fluorescence was quantified using ImageJ software. Sampling of cells was performed randomly. About 100 cells (from three wells) were scored per condition per experiment.

Cholesterol efflux measurements were carried out as described for dxr efflux except that cells were incubated with 10 µM BODIPY-cholesterol, a fluorescent derivative of cholesterol, and images were acquired using a 40×/1.3 Plan NeoFluar objective and filters for FITC.

### 4.9. Microscale Thermophoresis

Microscale thermophoresis (MST) is a biophysical technique that measures the strength of the interaction between two molecules by detecting a variation in the fluorescence signal of a fluorescently labeled target as a result of an IR-laser induced temperature change. The range of the variation in the fluorescence signal correlates with the binding of a ligand to the fluorescent target.

Membranes from yeast expressing human Ptch1 or human Smoothened were incubated at 30 µg/mL with 20 nM of the fluorescent dye NT-647 2nd gen (NanoTemper Technologies, München, Germany) to label the His-tag present at the c-terminus of both proteins. In this MST experiment, we kept the concentration of labeled membranes constant, while the concentration of non-labeled sPAH was varied between 250 µM and 15 nM. The assay was performed in PBS containing 0.5% DMSO. After a short incubation, the samples were loaded into Monolith™ NT.115 standard treated capillaries from NanoTemper Technologies and the MST analysis was performed using the NanoTemper Technologies Monolith NT.115 (LED: 30%; MST: medium). The fluorescence within the capillary is excited and detected through the same objective. A focused infrared laser is used to locally heat a defined sample volume. The MST response of fluorescent proteins within the temperature gradient is detected. After activation of the IR laser, a decrease in fluorescence is observed which corresponds to temperature-related intensity change (TRIC) triggered by the fast temperature change and thermophoretic movement of the fluorescent proteins out of the heated sample volume. The MST signal of fluorescent proteins changes or not upon binding to sPAH resulting in different MST traces. For analysis, the change in MST signal is expressed as the change in the normalized fluorescence (Fnorm), which is defined as F1/F0. Titration of sPAH results in a gradual change in MST signal, which is plotted as Fnorm against the sPAH concentration to yield a dose–response curve, which has been fitted to derive binding constants (Kd). The sPAH Kd was determined for 3 independent experiments.

### 4.10. In Silico Docking

Docking of vemurafenib, doxorubicin, and PAH on Ptch1 structure were performed using Vina toolkit [44] in USCF Chimera [45]. The structure of Ptch1 was obtained from RCSB Protein Data Bank (PDB ID: 6N7H, chain A) [23] and prepared using USCF Chimera Predock Toolkit. For the missing side chains, the Dunbrack rotamer 2010 library [46] was used and charges were assigned with ANTECHAMBER Amber ff14SB force field [47]. The docking was first done on the whole protein structure. Observing that the poses with lowest scores were in the previously predicted biologically relevant binding sites for cholesterol, docking was then performed by targeting the central cholesterol cavity.

The analysis was performed on the 10 best poses of the docking (ranked by score). Every interaction was compared to the cholesterol equivalents. Amino acids within a radius of 6 Å from the cholesterol were selected and listed in Table 2. We highlighted amino acid mutations due to single nucleotide variation associated to disease (with a probability above 0.8) which were reported in BioMuta database [48].

For the docked ligands, interactions were further analyzed using PoseView [49], a function of the ProteinsPlus web server [50]. In order to assess conserved amino acids among the proteins from Patched family, sequence alignment of Patched family members was performed on 30 sequences using T-COFFEE server for transmembrane proteins [51].

### 4.11. Quantification of Metabolic Stability

A 10 µL aliquot of the sPAH stock solution (10 mM in DMSO) was diluted in 990 µL of a mixture of acetonitrile and water. This solution was then diluted 100-fold in phosphate buffer containing mice liver microsomes (0.5 mg/mL), 1 mM NADPH, and 3 mM MgCl_2_, and incubated at 37 °C. After 2, 10, 20, 40, and 60 min, 70 µL aliquots were collected and mixed with 70 µL acetonitrile at 0 °C. Equivalent experiments were performed without NADPH in order to identify chemical instability or enzymatic process not depending on NADPH, and with testosterone as a positive control. The enzymatic reaction was stopped by addition of acetonitrile. Samples were analyzed by LC–MS/MS on an UHPLC LC–MS 8030 (Shimazu, Kyoto, Japan).

For metabolite identification, 50 µM of sPAH were incubated with mice liver microsomes and NADPH. Two samples were prepared: one in which acetonitrile was added immediately (t0) and one in which acetonitrile was added after 30 min. After centrifugation for 5 min at 15,000× *g*, 2 samples of each supernatant were analyzed by LC–MS/MS on a LC–MS 8030 (Shimazu, Kyoto, Japan) and detected by selected ion monitoring (SIM).

### 4.12. sPAH Toxicity on Chick Embryos

These experiments were performed by the company INOVOTION (La Tronche, France). Fertilized White Leghorn eggs were incubated at 37.5 °C with 50% relative humidity for 9 days. At this time (E9), an access point to the chorioallantoic membrane (CAM) was made by drilling a small hole through the eggshell into the air sac and a 1 cm^2^ window was cut in the eggshell above the CAM. Twenty-one eggs were used for each condition. Because of some instances of early death just after the opening of the shell (surgical act), data could be collected in fewer than 21 eggs per group. MeWo cells cultivated in DMEM medium with 10% FBS (and 1% penicillin/streptomycin) were detached with trypsin, washed with complete medium, labeled, and suspended in PBS. An inoculum of 3 × 10^6^ cells was added onto the CAM of each egg (E9). Eggs were then randomized in 4 groups. The chick embryos were then treated every two days (E11, E13, E15, E17) for 10 days in total, by adding 100 µL of vehicle (1% DMSO in PBS) as a negative control or 100 µL of sPAH at 3 different doses onto the CAM. The number of dead embryos evaluates the toxicity after 10 days of the treatment, as does the observation of 22 abnormality checkpoints in surviving embryos.

### 4.13. Effect of sPAH and Doxorubicin on Melanoma Cells Grafted in Chick Eggs

These experiments were performed by the company INOVOTION (La Tronche, France). Fertilized White Leghorn eggs were randomized in 4 groups 9 days after inoculation of 3.10^6^ MeWo cells onto the CAM of each egg. At day 10 (E10), tumors began to be detectable and they were treated by adding 100 µL of vehicle (1.2% DMSO in PBS), doxorubicin, and sPAH alone or with doxorubicin. Eggs were treated every two days (E10, E12, E14, E16, E18) for 10 days in the same way. At day 19 (E19), the upper portion of the CAM was removed, washed in PBS, and directly transferred in PFA for 48 h, and the tumors were then carefully cut away from normal CAM tissue. Tumors were weighed, and one-way ANOVA analysis with post-tests was performed on these data.

### 4.14. Preparation and Characterization of i-Particles Loaded with sPAH

iP-sPAH synthesis was custom-developed by Adjuvatis (Lyon, France) based on i-Particles^®^ preparation by nanoprecipitation method [52], using poly(d,l-lactic acid) (PLA) as polymer. No surfactant or stabilizer was required to stabilize the colloid solution. The resulted 180 nm size iP-sPAH were precisely characterized for their physicochemical parameters (hydrodynamic diameter, size distribution, and zeta potential), using a zetasizer NanoZS from Malvern-Panalytical (UK) and after high dilution in 0.22 µm-filtered 1 mM NaCl solution. sPAH loading (%DL) and entrapment efficiency (%EE) within i-Particles were also precisely quantified by fluorimetry after PLA and sPAH solubilization in acetonitrile, using a standard curve of sPAH ranging from 5 to 55 µg/mL in the same solvent. The measurements were made using a fluorimeter equipped with a 96-well microplate reader (infinite M1000, Tecan, Mannedorf, Switzerland) and black microplates (Greiner Bio-One, Courtaboeuf, FranceCity) at 288 nm excitation and 324 nm emission. The DL and EE were calculated by following equations:

$$\mathrm{DL}\;\left(\%\right)=\frac{\mathrm{encapsulated}\;\mathrm{mass}\;\mathrm{of}\;\mathrm{PAH}}{\mathrm{total}\;\mathrm{mass}\;\mathrm{of}\;\mathrm{the}\;\mathrm{particles}}\times 100$$

$$E\;\left(\%\right)=\frac{\mathrm{encapsulated}\;\mathrm{mass}\;\mathrm{of}\;\mathrm{PAH}}{\mathrm{total}\;\mathrm{mass}\;\mathrm{of}\;\mathrm{PAH}\;\mathrm{in}\;\mathrm{the}\;\mathrm{formulation}}\times 100$$

### 4.15. Evaluation of the Toxicity of sPAH in Mice

sPAH encapsulated in i-Particles was diluted in saline solution and injected intraperitoneally at 40, 13.3, 4, and 1.33 mg/kg (10 mL/kg) in Swiss (CD-1) mice (2 mice per dose). Monitoring and evaluation of toxicity was carried out for 72 h based on the behavioral signs and appearance of the animals. All experiments were carried out following protocols approved by the ethics committee from the University of Strasbourg (APAFIS#3671-2016012012046243) and in accordance with the council of the European communities’ guidelines for animal studies (86/609/CEE).

### 4.16. Melanoma Xenograft Study in Mice

Forty-eight male athymic Nu/NU NMRI mice (4–5 weeks) were purchased from Charles River and housed under pathogen-free conditions. A 200 µL aliquot of 3 Millions A375 BRAF^V600E^ melanoma cells were injected subcutaneously per mouse. Tumor-bearing mice were treated with 4 therapeutic cycles, which consisted of either vemurafenib (8 mg/kg body weight (5 µL/g in olive oil) intraperitoneally 5 days a week) plus empty i-Particles (lot 180509-DF-CP02, 4 µL/g intraperitoneally every other day), iP-sPAH (Lot: 180821-DF-PAH, 5 mg/kg body weight (4 µL/g) intraperitoneally every other day), or a combination of vemurafenib and iP-sPAH (vemurafenib 8 mg/kg body weight intraperitoneally every other day plus iP-sPAH 5 mg/kg body weight intraperitoneally 5 days a week). Mice from the control group were injected with olive oil (5 µL/g) intraperitoneally 5 days a week plus empty i-Particles (lot 180509-DF-CP02, 4 µL/g) intraperitoneally every other day. Animals were monitored daily for symptoms of disease (weight loss >20%, ruffled coat, hunched back, weakness, reduced motility) and tumor sizes were measured every 3 to 4 days. At day 23 after the start of treatment, when the first tumors reached the longest tumor diameter of 1.5 cm, the study was terminated, and animals sacrificed. Tumors were excised and subsequently snap frozen in isopentane using OCT (optimal cutting temperature) compound. This study was conducted in agreement with the French Guidelines for animal handling and approved by local ethics committee (APAFIS#18382-201901081114131v3), and in accordance with the council of the European communities’ guidelines for animal studies.

A part of each tumor was cut in 8 µm thick tissue sections using a microtome-cryostat for quantification of the number of apoptotic tumor cells and of proliferative cells performed using the colorimetric DeadEND TUNEL System (Promega, Mannheim, Germany) and immunofluorescence analysis of Ki67 (BD pharmigen 556003), respectively. OCT was removed from the remainder of each tumor, and tumors were crushed in 50 mM Tris-HCl pH 6.8, 10% glycerol, and 2% SDS), heated at 95 °C, and sonicated for Western blot analyses as well as vemurafenib and cholesterol quantification. Protein concentration in each homogenate was evaluated using a Bio-Rad protein assay based on the Bradford dye-binding method.

### 4.17. Quantification of Vemurafenib in Tumors

Metabolites were extracted from tumor homogenates using methanol and resuspended in 40% acetonitrile before mass spectrometry analysis. Briefly, the metabolites were separated with UPLC system (ThermoFisher, Illkirch, France on a C18 column in an appropriate gradient. Mass spectrometry data were acquired with a Q-Exactive *plus* mass spectrometer (ThermoFisher) operating in Parallel Reaction Monitoring (PRM) mode. Finally, vemurafenib was identified using Xcalibur Quan-Browser software version 4.1.31.9 (ThermoFisher).

### 4.18. Quantification of Cholesterol in Tumors

Total lipids were extracted from the tumor homogenates according to the method developed by Folch [53]. The total lipids were submitted to alkaline hydrolysis in 5 mL of 0.35 M KOH for 2 h at ambient temperature. The solution was neutralized with 65 µL of phosphoric acid, and the sterols were extracted with 9 mL chloroform in the presence of 3 mL 0.9% sodium chloride. The organic phase was removed, and the solvent was evaporated to dryness. For the quantification of sterols, 5 α-cholestane was added as an internal standard. The samples were derivatized to trimethylsilyl ethers by heating at 60 °C for 30 min after the addition of 100 µL of pyridine and 100 µL of BSTFA (Supelco, Bellafonte, PA, USA). The derivatives were analyzed on a gas chromatograph coupled to a flame ionization detector (GC–FID Hewlett-Packard HP5890A). A 1 µL aliquot was introduced by automated injection in splitless mode at 290 °C on a DB-5MS fused silica capillary column (30 m × 0.25 mm id, 0.25 µm film thickness; J&W Scientific, Agilent Technologies, Massy, France). The initial oven temperature was kept at 60 °C for 1 min, then increased at a rate of 20 °C/min to 290 °C, and then 2 °C/min to a final temperature of 300 °C.

### 4.19. SDS-PAGE and Western Blotting

Total RIPA extracts from cells or tumor homogenates were prepared. Protein concentrations were determined by the DC Protein Assay (Bio-Rad, Marnes-la-Coquette, France). Samples (50 to 80 µg) were separated on SDS-PAGE and transferred to nitrocellulose membranes (Amersham, Bath, UK) using standard techniques. After 1 h at room temperature in blocking buffer (20 mmol/L Tris-HCl pH 7.5, 45 mmol/L NaCl, 0.1% Tween-20, and 5% non-fat milk), nitrocellulose membranes were incubated overnight at 4 °C with rabbit anti-Patched antibody (Abcam ab53715; 1/1000), rabbit anti-Phospho-Erk1/2 antibody (Cell Signaling Technology (Leiden, The Netherlands); 1/1000), rabbit anti-Erk1/2 antibody (Cell Signaling Technology; 1/1000), or mouse anti-β-tubulin antibody (Sigma; 1/1000). After 3 washes, membranes were incubated for 45 min with anti-rabbit (1:2000) or anti-mouse (1:5000) immunoglobulin coupled to horseradish peroxidase (Dako-Agilent, Santa Clara, CA, USA). Detection was carried out with an ECL Prime Western blotting detection reagent (Amersham) on a Fusion FX imager (Vilber Lourmat, Collegien, France), and analyses were performed using ImageJ software.

### 4.20. Statistical Analysis

All results represent at least three independent replications. Data are shown as mean value ± SEM. Prism 6 (GraphPad) was used to determine IC_50_ values and other statistical analyses using one-way analysis of variance (ANOVA) followed by Bonferroni’s multiple comparison tests.

## 5. Conclusions

Altogether, our data show that panicein A hydroquinone is able to increase vemurafenib effectiveness against resistant BRAF^V600E^ melanoma cells and to eliminate resistant melanoma cells without undesirable side effects. sPAH specifically binds to Ptch1 and inhibits the Ptch1 drug efflux activity both in vitro and in vivo after encapsulation to protect it from oxidation. SAR studies allowed us to identify one of the key pharmacophores responsible for this activity, and a new chemical synthesis pathway under development by our team makes it possible to consider the rapid preparation of large number of PAH analogues in order to obtain a more stable and efficient lead and a potential drug candidate. We conclude that sPAH is a very promising lead for vemurafenib resistant BRAF^V600E^ melanoma where Ptch1 is overexpressed.

## 6. Patent

Mus-Veteau I, Thomas O, Tribalat MA, Azoulay S. (27/10/2014, PCT/EP2015/074771). Patched inhibiting compounds, composition and uses thereof. Delivered in USA in April 2019 and in Japon in January 2020.

## Figures and Tables

**Table 1:** Synthetic panicein A hydroquinone (sPAH) increases doxorubicin cytotoxicity against several melanoma cell lines. IC_50_s were calculated using GraphPad Prism 6 software. The mean ± SEM of 3 independent experiments are presented.

Treatment	Dxr IC_50_ (µM)		
	MeWo	MeWo DxrR	A375
Dxr + DMSO	141.5 ± 2.2	247 ± 49	41 ± 8.2
Dxr + sPAH 20 µM	1.1 ± 0.4	1.75 ± 1	0.6 ± 0.2

**Table 2:** Synthetic PAH is as effective as natural PAH to increase dxr cytotoxicity against melanoma cells. IC_50_ values were calculated using GraphPad Prism 6 software. Mean ± SEM of 3 independent experiments are presented.

Heading Treatment		IC_50_ (µM)	
		With Natural PAH	With Synthetic PAH
MeWo cells	Dxr (at 20 µM PAH)	0.5 ± 0.1	1.1 ± 0.4
	PAH at 2 µM Dxr	6.6 ± 0.8	8.1 ± 1.3
A375 cells	Dxr (at 20 µM PAH)	0.5 ± 0.1	0.6 ± 0.2
	PAH at 2 µM Dxr	12.4 ± 4.9	12.4 ± 2.3

**Table 3:** List of amino acids within a radius of 6Å of a ligand in the best docking pose.

Ligand	Amino Acids Involved
**Cholesterol**	V125, E126, L**128**, W129, *L427*, *L431*, F434, N496, *A497*, A498, T**499**, V502, *I567*, *A569*, L570, *F573*, L775, D**776**, L777, I780, Q794, Y801, F987, Y1013, F1017, W**1018**, Q**1020**, S1079, V**1081**, F1147, I1148, Y1151, F**1152**
**Dxr**	N124, *V125*, *L**128***, *W129*, F422, T424, *L427*, L*431*, G774, *L775*, ***D776***, *L777*, *F1017*, *W**1018***, Y1021, S1079
**Vemurafenib**	*V125*, *W129*, *N496*, *A497*, *A498*, *A569*, *L775*, *D**776***, *L777*, *I780*, *Y801*, *Y1013*, *F1017*
**PAH**	*V125*, *W129*, *F434*, *N496*, *A497*, *A498*, *A569*, *L570*, *L775*, *D**776***, *L777*, *I780*, *F1017*, *W**1018***

Underlined amino acids have side chains orientated toward the cholesterol or interactions, found by PoseView, with dxr, vemu, and PAH. Amino acids with mutations that result in a damaging phenotype are indicated in **bold**, between *asterisks* when conserved in the family, and in *italics* if common with an amino acid listed for the cholesterol.

## Supplementary Materials

The following are available online at https://www.mdpi.com/2072-6694/12/6/1500/s1, Figure S1: Effect of PAH precursors and analogues on dxr cytotoxicity in A375 cells. Cell viability was measured after treatment with increasing concentration of dxr with DMSO or 20 µM of sPAH. sPAH precursors 8 or 9, or sPAH analogues 11, 12, or 13 in A375 cells. Dxr-IC_50_ values calculated in the presence of sPAH or sPAH analogue 13 are presented, Figure S2: sPAH increases cisplatin cytotoxicity against MeWo and A375 melanoma cell lines. Cells were treated with increasing concentrations of cisplatin in the presence of DMSO or sPAH 20 µM. IC_50_ values were calculated using GraphPad Prism 6 software. The mean ± SEM of at least 3 experiments are presented, Figure S3: Cholesterol binding pocket on Ptch1 structure. Chain A of pdb6n7h with cholesterol (magenta). Amino acids underlined in Table 3 are represented as sticks, Figure S4: sPAH is oxidized into its quinone form upon contact with liver microsomes. For metabolite identification, 50 μM of sPAH were incubated with mice liver microsomes and NADPH. Two samples were prepared: one in which acetonitrile was added immediately (t0) and one in which acetonitrile was added after 30 min. Samples were analyzed by LC–MS/MS and detected by selected ion monitoring (SIM). LC–MS/MS analysis shows that sPAHis oxidized in its quinone form (9), Figure S5. PAH-loaded i-Particles size distribution and colloidal stability. Particles size distribution and colloidal stability were determined by dynamic light scattering using a Zetasizer Nano ZSP from Malvern-Panalytical. In a typical experiment, 20 μL of dispersions were added to 1.5 mL of 0.22 μm-filtered 1 mM NaCl solution. A laser of wavelength 633 nm was used as source and detector was placed at a 173° angle. Measurements were carried-out at 25 °C. Zeta potential was determined by measuring electrophoretic mobility in a 0.22 μm-filtered 1 mM NaCl solution using a Zetasizer Nano ZSP from Malvern-Panalytical and a disposable folded capillary cell. A laser of wavelength 633 nm was used as source and detector was placed at a 13° angle. Measurements were carried-out at 25 °C. A. The hydrodynamic diameter (average size (Z-average) and polydispersity index (PdI) based on DLS), zeta potential (mV), drug loading (DL%), and encapsulation efficiency (EE%) of PAH-loaded i-Particles. Mean and SD of 4 measurements. B. Variation of hydrodynamic diameter (Z-average based on DLS, filled square and polydispersity index, PdI, empty squares) of iP-PAH (drug loading 8.49%) with storage time (+4 °C, fridge). Four measurements were taken, and the averages and standard deviations are presented, Figure S6: The addition of iP-sPAH to vemurafenib more strongly inhibits ERK phosphorylation in tumors. Tumor extracts were analyzed by Western blotting with anti-Phospho-Erk1/2 and anti-Erk1/2 antibodies. Signal quantification was performed using ImageJ software and the pERK/ERK ratio was calculated and reported for each tumor extract. Significance is attained at *p* < 0.05 (*), ns: no significant difference, Figure S7: (**A**) Whole Western blot of Figure 1B. The membrane was cut into 2 pieces incubated respectively with anti-Ptch1antibody and anti-b tubulin antibody. (**B**) Whole Western blot of Figure 1C. V The membrane was cut into 2 pieces incubated respectively with anti-Ptch1antibody and anti-b tubulin antibody. (**C**) Whole Western blot of Supplementary Figure 6 and densitometry of each band. The membrane was incubated first with anti-phosphoERK antibody, then stripped and incubated with ERK antibody and then with anti-btubulin antibody. Densitometry of each band is shown in the table.
