# Supplementary material for: Inhibition of Patched Drug Efflux Increases Vemurafenib Effectiveness against Resistant BrafV600E Melanoma
Source: Cancers (Basel). 2020 Jun 9;12(6):1500. doi: 10.3390/cancers12061500 (PMC7352342; doi:10.3390/cancers12061500)
Supplement: Supplementary file 1 [file cancers-12-01500-s001.pdf]

cancers

MDPI

Article

Inhibition of Patched Drug Efflux Increases Vemurafenib Effectiveness against Resistant Braf^{V600E} Melanoma

Laurie Signetti [1], Nelli Elizarov [2], Méliné Simsir [1], Agnès Paquet [1], Dominique Douguet [1], Fabien Labbal [1], Delphine Debayle [1], Audrey Di Giorgio [2], Valérie Biou [3], Christophe Girard [4], Maria Duca [2], Lionel Bretillon [5], Corine Bertolotto [4], Bernard Verrier [6], Stéphane Azoulay [2*], and Isabelle Mus-Veteau [1*]

[1] Université Côte d'Azur, CNRS, IPMC, 660 Route des Lucioles, 06560 Valobonne, France; lauriesignetti@gmail.com (L.S.); simsir@ipmc.cnrs.fr (M.S.); paquet@ipmc.cnrs.fr (A.P.); douguet@ipmc.cnrs.fr (D.D.); labbal@ipmc.cnrs.fr (F.L.); debayle@ipmc.cnrs.fr (D.D.)
[2] Université Côte d'Azur, CNRS, ICN, 28 Avenue Valrose, 06108 Nice, CEDEX 2, France; nelli.elizarov@yahoo.de (N.E.); Audrey.DI-GIORGIO@univ-cotedazur.fr (A.D.G.); Maria.DUCA@univ-cotedazur.fr (M.D.)
[3] Sorbonne Paris Cité, CNRS, IBPC, Laboratoire de Biologie Physico-Chimique des Protéines Membranaires, Institut de Biologie Physico-Chimique, University Paris Diderot, 13 rue Pierre et Marie Curie, 75005 Paris, France; valerie.biou@ibpc.fr
[4] Université Côte d'Azur, INSERM, CNRS, C3M, Bâtiment Universitaire ARCHIMED 151 Route Saint Antoine de Ginestière BP 2 3194, 06204 Nice, CEDEX 3, France; christophe.girard@univ-cotedazur.fr (C.G.); Corine.Bertolotto@unice.fr (C.B.)
[5] Centre des Sciences du Goût et de l'Alimentation, Université Bourgogne Franche-Comté CNRS, INRA, SSGA, AgroSup Dijon, F-21000 Dijon, France; lionel.bretillon@dijon.inra.fr
[6] Adjuvatis SAS, IBCP, 7 passage du Vercors—69007 Lyon, France; bernard.verrier@adjuvatis.com
* Correspondence: stephane.azoulay@univ-cotedazur.fr (S.A.); mus-veteau@ipmc.cnrs.fr (I.M.-V.)

Supplementary

Chemical synthesis of Panicein A Hydroquinone based on the procedure of Davis and al.[i]

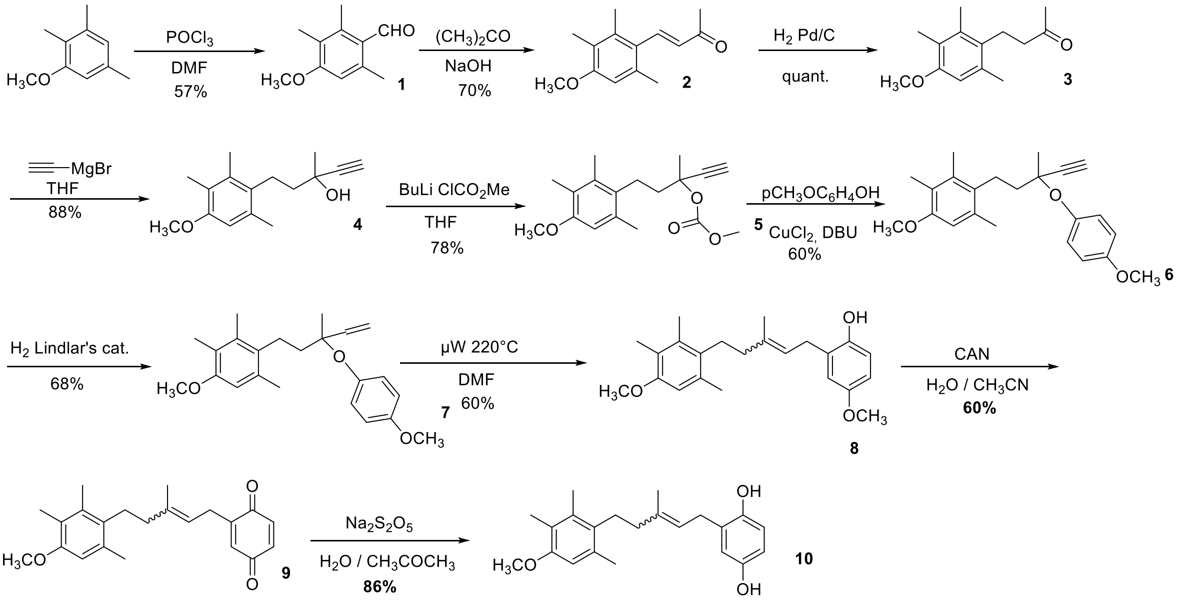

Compound **1**: Under an Ar-atmosphere phosphorus oxychloride (4.1 mL, 1.2 eq) was added dropwise with stirring to dry DMF (8 mL) at 10-20°C during 15 min, followed by the addition of 2,3,5-trimethylanisole (5 g) followed. The mixture obtained was heated to 110°C overnight. The mixture is then poured into a NaOAc/water/ice solution and stirred for 1h at ambient temperature. The aqueous layer was extracted with ethyl

acetate. After the extraction, the combined organic extract was washed with 1.5% HCl, water, dried over Na_2SO_4, filtered and the solvent was removed in vacuum. The crude residue was purified by flash chromatography on silica gel using a gradient of Cy/EA (12:1) as the eluent to give **1** as a light yellow solid. Yield=57%. R_f= 0.424 (Cy/EA 6:1). ^1H ($CDCl_3$, 200 MHz) δ 10.51 (s, 1H, 1-H), 6.55 (s, 1H, 5-H), 3.86 (s, 3H, 4-H), 2.59 (s, 3H, 3/2/6-H), 2.51 (s, 3H, 3/2/6-H), 2.13 (s, 3H, 3/2/6-H).

Compound **2**: **1** (6.91 g) was dissolved in acetone (93 mL) and water (37 ml). Then 10% NaOH (19.4 ml) was added dropwise at 0-5°C over 30 min. The reaction mixture was stirred at ambient temperature overnight, acidified to pH 4-5 with AcOH, and the solvent removed in vacuum. The residue was extracted with ethyl acetate, the organic phase washed with 5 % sodium bicarbonate solution, water, dried over Na_2SO_4, filtered and the solvent was removed in vacuum. The crude residue was purified by flash chromatography on silica gel using a gradient of Cy/EA (30:1) as the eluent to give **2** as a light yellow solid. Yield=68 %. R_f= 0.324 (Cy/EA 8:1).^1H ($CDCl_3$, 200 MHz) δ 7.70 (d, *J*= 16.5 Hz, 1H, 2/3-H), 6.60 (s, 1H, 7-H), 6.24 (d, *J*= 16.5 Hz, 1H, 2/3-H), 3.83 (s, 3H, 6-H), 2.39 (s, 3H, 1-H), 2.34 (s, 3H, 4/5/8-H), 2.26 (s, 3H, 4/5/8-H), 2.15 (s, 3H, 4/5/8-H). ^{13}C NMR (101 MHz, $CDCl_3$) δ 200.7, 198.7, 157.6, 157.1, 143.2, 142.6, 136.8, 135.0, 134.76, 132.8, 132.7, 132.6, 128.2, 126.8, 123.4, 122.9, 110.3, 109.8, 55.6, 55.6, 29.5, 27.5, 21.7, 20.9, 17.6, 17.4, 11.9, 11.8.

Compound **3**: Compound **2** was dissolved in ethylacetate (10 mL solvent for 1 mmol of starting material). The 1-2mol% Pd/C (10 mol%) was added and the reaction mixture was stirred overnight under hydrogen atmosphere. The crude residue was purified by flash chromatography on silica gel using a gradient of PE/EA (5:1) as the eluent to give **13** as a white powder. Yield=96%. R_f= 0.359 (Cy/EA 5:1). ^1H ($CDCl_3$, 200 MHz) δ 6.57 (s, 1H, 7-H), 3.79 (s, 3H, 6-H), 2.90-2.80 (m, 2H, 2/3-H), 2.62-2.53 (m, 2H, 2/3-H), 2.30 (s, 3H, 1-H), 2.20 (s, 3H, 1/4/5/8-H), 2.18 (s, 3H, 1/4/5/8-H), 2.14 (s, 3H, 1/4/5/8-H). ^{13}C ($CDCl_3$, 200 MHz) δ 155.6, 135.9, 133.7, 129.6, 123.1, 110.5, 55.6, 43.5, 29.9, 23.7, 20.4, 15.8, 12.0.

Compound **4**: Under an argon atmosphere **3** (1g) was dissolved in dry THF and the mixture was cooled with ice/NaCl to -20°C. Ethynylmagnesium bromide (11mL, 1.2 eq) was added drop wise. The reaction mixture was stirred at ambient temperature overnight, quenched with a saturated NH_4Cl solution and extracted with ethyl acetate. The organic phase was dried over Na_2SO_4 and the solvent was removed under pressure. The crude residue was purified by flash chromatography on silica gel using a gradient of Cy/EA (10:1) as the eluent to give **4** as a white solid. Yield= 76%. R_f= 0.307 (Cy/EA 5:1). ^1H NMR (200 MHz, $CDCl_3$) δ 6.58 (s, 1H, 9-H), 3.80 (s, 3H, 8-H), 2.95 – 2.72 (m, 2H, 5-H), 2.54 (s, 1H, 3-H), 2.35 (s, 3H, 7/6/10-H), 2.26 (s, 3H, 7/6/10-H), 2.15 (s, 3H, 7/6/10-H), 1.88 – 1.67 (m, 2H, 4-H), 1.59 (s, 3H, 2-H). ^{13}C NMR (50 MHz, $CDCl_3$) δ 155.5, 136.1, 133.9, 130.2, 123.0, 110.5, 87.5, 71.8, 68.1, 55.7, 42.9, 29.9, 24.8, 20.4, 15.8, 12.1.

Compound **5**: To a solution of compound **4** (1.0 g) in THF at 0°C, dropwise n-butyllithium (2.5 M in hexane; 2.25 mL, 1.1 eq) was added. The mixture was stirred at this temperature for 30 min and methyl chloroformate (379 μL, 1.2 eq) was then added dropwise. The reaction mixture was allowed to warm to room temperature and stirred overnight. An extraction with ethyl acetate followed. The organic phase was dried over Na_2SO_4 and the solvent was removed under vacuum. The crude residue was purified by flash chromatography on silica gel using a gradient of Cy/EA (15:1) as the eluent to give **5** as a white solid. Yield=78 %. R_f= 0.42 (Cy/EA 5:1). ^1H NMR (200 MHz, $CDCl_3$) δ 6.58 (s, 1H, 9-H), 3.81 (s, 3H, 8-H/3-H), 3.80 (s, 3H, 8-H/3-H), 2.94 – 2.78 (m, 2H, 5-H), 2.70 (s, 1H, 1-H), 2.35 (s, 3H, 7/6/10-H), 2.26 (s, 3H, 7/6/10-H), 2.16 (s, 3H, 7/6/10-H), 2.13 – 1.85 (m, 2H, 4-H), 1.82 (s, 3H, 2-H). ^{13}C NMR (126 MHz, $CDCl_3$) δ 155.6, 153.8, 136.1, 133.9, 129.6, 123.0, 110.5, 83.1, 77.2, 76.9, 74.2, 55.6, 54.5, 40.9, 26.3, 24.3, 20.3, 15.7, 12.0.

Compound **6**: To a solution of 4-methoxyphenol (1.9 g), anhydrous copper(II) chloride (2.6 mg, 1.0 mol%) in acetonitrile (4 mL) at 0°C, 1,8-diazabicyclo[5.4.0]undéc-7-ène (DBU) (317 µL, 1.1 eq) was dropwise added. The mixture was stirred for 15 min and a solution of **5** (1.2 eq) in acetonitrile (6mL) was then added dropwise at 0°C. The reaction mixture was stirred overnight, then quenched with water and extracted with ethyl acetate. The combined organic extracts were dried and concentrated. The crude residue was purified by flash chromatography on silica gel using a gradient of PE/EA (30:1) as the eluent to give **6** as a white solid. Yield=66%. R_f= 0.275 (PE/EA 45:1).^{1}H NMR (500 MHz, $CDCl_3$) δ 7.25 – 7.20 (m, 2H, 10-H, 10'-H), 6.92 – 6.85 (m, 2H, 11-H, 11'-H), 6.63 (s, 1H, 8-H), 3.85 (s, 3H, 7-H/12-H), 3.84 (s, 3H, 12-H/7-H), 3.10 – 2.90 (m, 2H, 4-H), 2.69 (s, 1H, 1-H), 2.41 (s, 3H, 5/6/9-H), 2.32 (s, 3H, 5/6/9-H), 2.22 (s, 3H, 5/6/9-H), 2.13 – 1.92 (m, 2H, 3-H), 1.67 (s, 3H, 2-H). ^{13}C NMR (126 MHz, $CDCl_3$) δ 155.8, 155.5, 149.2, 136.3, 134.0, 130.4, 123.5, 122.9, 114.0, 110.5, 85.4, 77.2, 75.9, 75.2, 55.7, 42.2, 27.1, 27.0, 24.8, 20.4, 15.8, 12.1.

Compound **7**: Compound **6** was dissolved in MeOH (10 mL solvent for 1 mmol of starting material). The Lindlar catalyst (20mg catalyst for 0.5 mmol of starting material) and quinoline (0.25 mL quinoline for 0.5 mmol of starting material) were added and the reaction mixture was stirred overnight under hydrogen atmosphere. The hydrogen passed directly into the reaction mixture. When the starting material was completely consumed, the mixture was filtered through Celite, washed with methanol, and concentrated in vacuo. The crude residue was purified by flash chromatography on silica gel using a gradient of PE/EA (20:1) as the eluent to give7 as a pale yellow oil. Yield=68%. R_f= 0.3 (Cy/EA 30:1).
^{1}H ($CDCl_3$, 200 MHz) *δ* ^{1}H NMR (200 MHz, $CDCl_3$) δ 6.97 (d, *J* = 9.0 Hz, 2H, 11-H, 11'-H), 6.78 (d, *J* = 8.9 Hz, 2H, 12-H, 12'-H), 6.57 (s, 1H, 9-H), 6.18 (dd, *J* = 17.4, 11.1 Hz, 1H, 2-H), 5.30 – 5.10 (m, 2H, 1-H), 3.79 (s, 3H, 8/13-H), 3.78 (s, 3H, 13/8-H), 2.73 (m (t-like, *J* = 8.3 Hz), 1H, 5-H), 2.29 (s, 3H, 6/7/10-H), 2.21 (s, 3H, 6/7/10-H), 2.15 (s, 3H, 6/7/10-H), 1.98 – 1.68 (m, 2H, 4-H), 1.45 (s, 3H, 3-H)

Compound **8**: A solution of compound **7** (400 mg) in DMF (4 mL) in a sealed tube was heated at 220°C for 20 min in a microwave reactor (300 W). The reaction mixture was then diluted with ethyl acetate (8 mL), the organics separated, and the aqueous layer further extracted with ethyl acetate (10 mL). The combined organic phases were then dried and concentrated in vacuo. The crude residue was purified by flash chromatography on silica gel using a gradient of PE/EA (5:1) as the eluent to give **8** as a white solid. Yield=96 %. R_f= 0.35 (PE/EA 5:1). ^{1}H NMR (400 MHz, $CDCl_3$) *δ* 6.80- 6.63 (m, 3H, 14-H, 11-H, 12-H), 6.60 (s, 1H, 5-H), 5.46 (t, *J* = 7.1 Hz) and 5.39 (t, *J* = 7.1 Hz) (1H, 9-H, *E* and *Z*), 5.01 (s, 1H, -OH), 3.82 (s, 1H, 13-H), 3.79 (s) and 3.78 (s) (1H, 1-H, *E* and *Z*), 3.41 (d, *J* = 7.1 Hz) and 3.36 (d, *J* = 7.1 Hz) (1H, 10-H, *E* and *Z*), 2.82 – 2.66 (m, 2H, 6-H), 2.38 (s) and 2.35 (s) (3H, 2-H/3-H, *E* and *Z*), 2.30 (s) and 2.26 (s) (3H, 3-H/2-H, *E* and *Z*), 2.37 – 2.14 (m, 2H, 7-H), 2.18 (s, 3H, 4-H), 1.93 (s) and 1.91 (s) (1H, 8-H, *E* and *Z*). ^{13}C NMR (101 MHz, $CDCl_3$) δ 155.5, 155.4, 153.7, 153.7, 148.3, 148.2, 138.6, 138.2, 136.0, 135.9, 133.7, 133.7, 130.9, 130.9, 128.3, 128.3, 122.9, 122.9, 122.6, 121.6, 116.3, 116.2, 115.8, 115.8, 112.1, 112.1, 110.5, 110.5, 77.2, 60.6, 55.8, 55.8, 55.7, 55.7, 39.7, 32.2, 29.7, 29.3, 28.9, 28.3, 23.7, 21.2, 20.5, 20.4, 16.5, 15.9, 15.8, 14.3, 12.1, 12.1.

Compound **9**: A solvent mixture of acetonitrile and water in a 2:1 ratio was prepared. 100 mg of **8** was dissolved in 2 mL of solvent and cooled to 0°C. Under stirring, CAN (2.2 eq) dissolved in 4 mL solvent was added dropwise to the mixture. The mixture was stirred for maximum 4h. The reaction was extracted with ethylacetate and the crude residue was purified by flash chromatography on silica gel using a gradient of PE/EA (20:1) as the eluent to give **9** as an orange oil. Yield=60 %. R_f= 0.436 (Cy/EA 5:1). ^{1}H NMR (400 MHz, $CDCl_3$) *δ* 6.83 – 6.62 (m, 2H, 11-H, 12-H), 6.62 – 6.37 (m, 2H, 13-H, 4-H), 5.24 (t, *J* = 7.1 Hz) and 5.19 (t, *J* = 7.5 Hz) (1H, 9-H E and Z), 3.80 (s) and

3.75 (s) (3H, 1-H, *E* and *Z*), 3.17 (d, *J* = 7.1 Hz) and 3.05 (d, *J* = 7.2 Hz) (2H, 10-H, *E* and *Z*), 2.78 – 2.59 (m, 2H, 6-H), 2.20-2.10 (m, 2H, 7-H), 2.32 (s) and 2.30 (s) (3H, 3/2/5-H, *E* and *Z*), 2.23 (s) and 2.21 (s) (3H, 3/2/5-H, *E* and *Z*) 2.15 (s) and 2.09 (s) (3H, 3/2/5-H, *E* and *Z*), 1.90 (s) and 1.75 (s) (3H, 8-H, *E* and *Z*). ^{13}C NMR (101 MHz, $CDCl_3$) δ 188.0, 187.9, 187.6, 155.5, 155.4, 148.5, 148.5, 140.5, 139.9, 136.9, 136.7, 136.5, 136.4, 136.1, 135.9, 133.9, 133.7, 132.5, 132.2, 130.8, 130.5, 123.0, 118.8, 117.9, 110.5, 110.5, 55.7, 55.5, 39.7, 31.9, 28.8, 27.9, 27.6, 27.3, 23.8, 20.6, 20.5, 16.5, 15.9, 15.9, 12.1, 12.1.

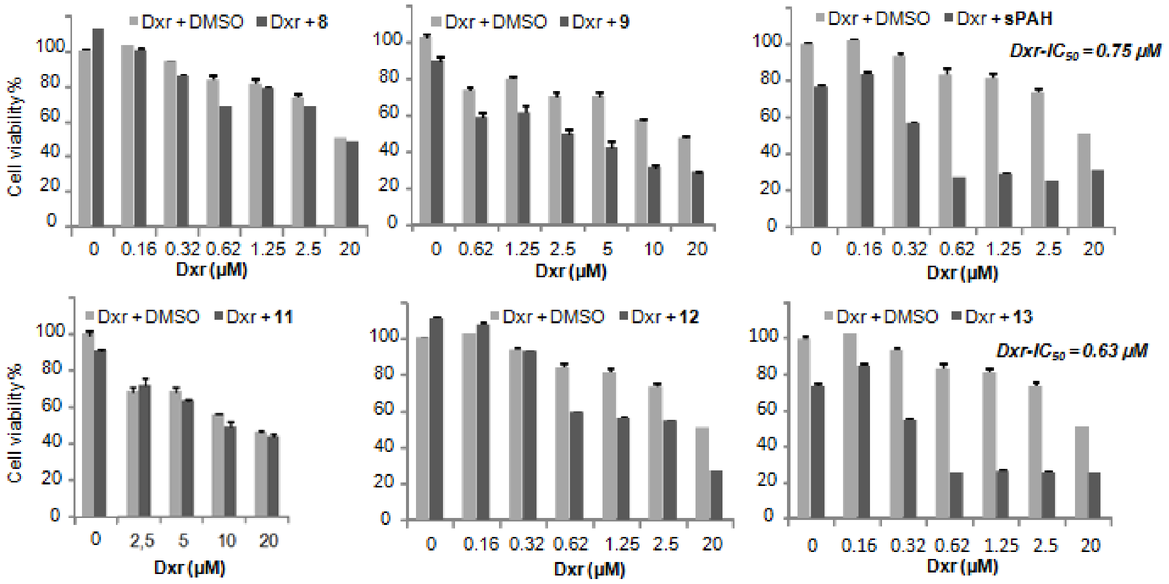

Figure S1. Effect of PAH precursors and analogues on dxr cytotoxicity in A375 cells. Cell viability was measured after treatment with increasing concentration of dxr with DMSO or 20 µM of sPAH. sPAH precursors 8 or 9, or sPAH analogues 11, 12 or 13 in A375 cells. Dxr -IC_{50} values calculated in the presence of sPAH or sPAH analogue 13 are presented.

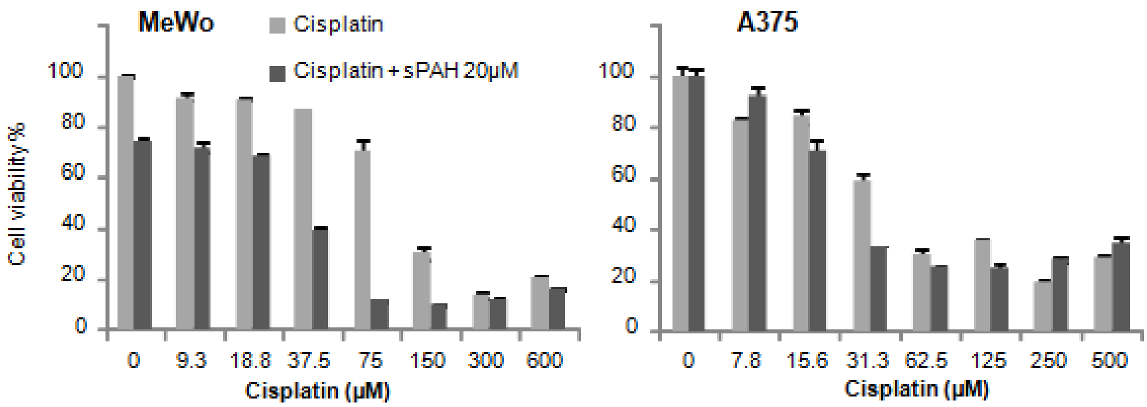

	Cisplatin-IC_{50} (µM)	
	MeWo	A375
+ DMSO	98.2 ± 11.7	33.4 ± 6.5
+ sPAH 20 µM	48,7 ± 9	17.1 ± 1.3

Figure S2. sPAH increases cisplatin cytotoxicity against MeWo and A375 melanoma cell lines. Cells were treated with increasing concentrations of cisplatin in the presence of DMSO or sPAH 20 µM.

IC_{50} were calculated using GraphPad Prism 6 software. The mean ±sem of at least 3 experiments are presented.

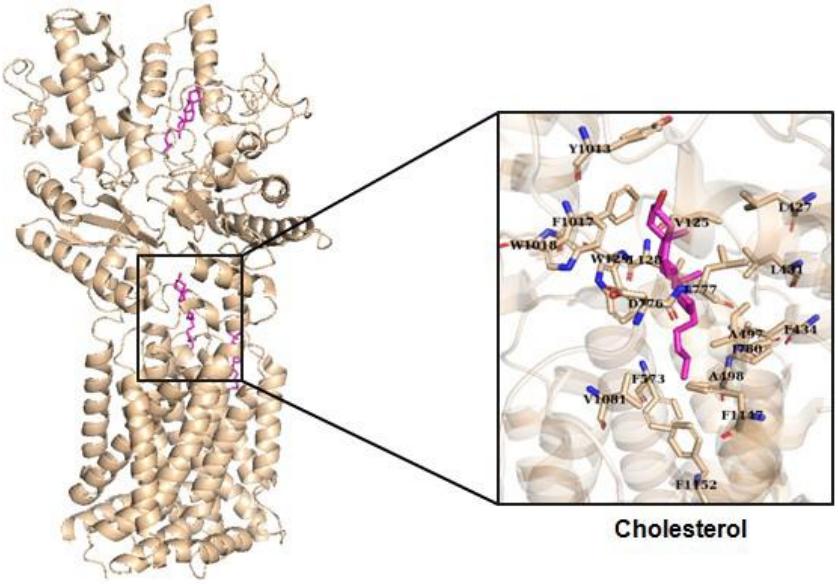

Figure S3. Cholesterol binding pocket on Ptch1 structure. Chain A of pdb 6n7h with cholesterol (magenta). Amino acids underlined in Table 3 are represented as sticks.

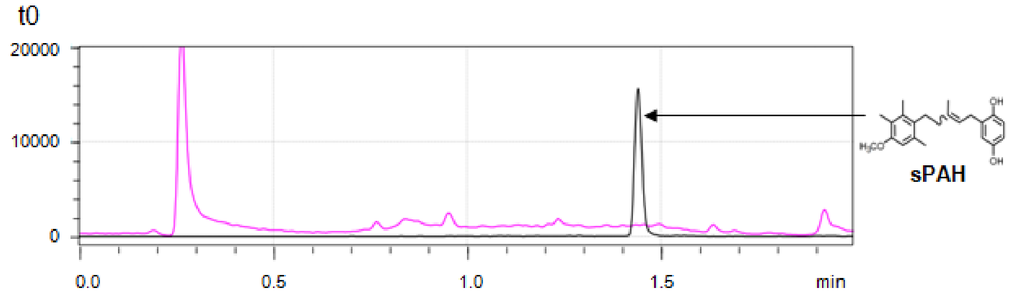

Peak at t_R=	Compound	Area
1.44 min	sPAH	20987

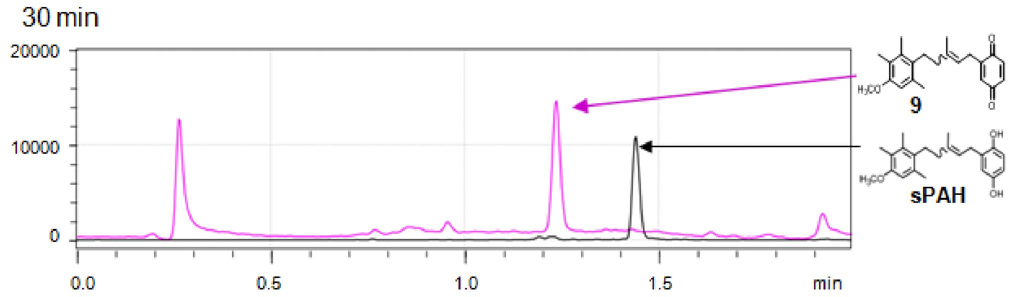

Peak at t_R=	Compound	Area	% remaining after 30 min
1.44 min	sPAH	14521	69%
1.24 min	Metabolite	20284	

Figure S4. sPAH is oxidized into its quinone form upon contact with liver microsomes. For metabolite identification, 50 µM of sPAH were incubated with mice liver microsomes and NADPH. Two samples were prepared: one in which acetonitrile was added immediately (t0) and one in which acetonitrile

was added after 30 min. Samples were analyzed by LC-MS/MS and detected by selected ion monitoring (SIM). LC-MS/MS analysis shows that sPAH is oxidized in its quinone form (9).

A

Z-average (nm)	PdI	Zeta Potential (mV)	DL %	EE %
185 ± 0.99 nm	0.020 ± 0.020	-55.5 ± 1.20 mV	1.20	100
181 ± 5.39 nm	0.035 ± 0.024	-57.9 ± 1.93 mV	4.32	92
185 ± 0.83 nm	0.020 ± 0.004	-61.2 ± 1.86 mV	8.49	79
188 ± 1.60 nm	0.022 ± 0.012	-56.9 ± 1.30 mV	9.72	97

B

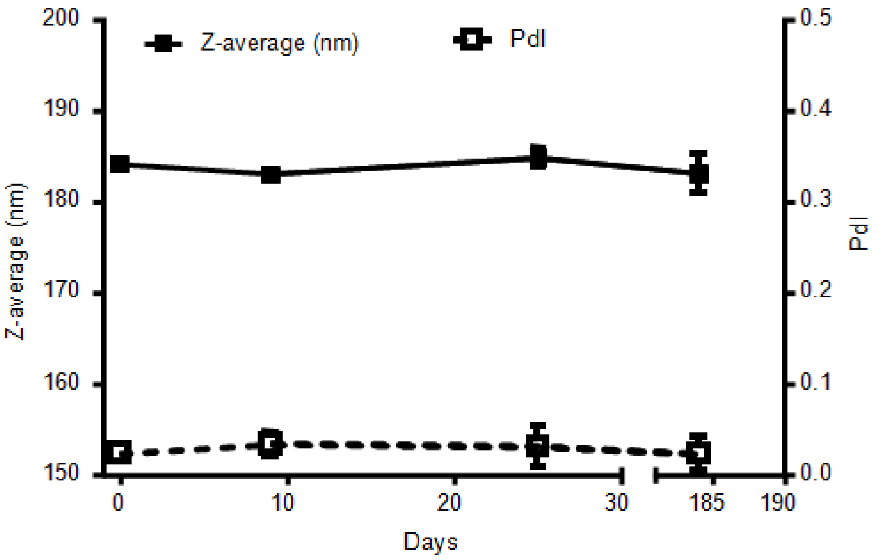

Figure 5. PAH-loaded i-Particles size distribution and colloidal stability. Particles size distribution and colloidal stability were determined by dynamic light scattering using a Zetasizer NanoZSP from Malvern-Panalytical. In a typical experiment, 20 µL of dispersions were added to 1.5 mL of 0.22 µm-filtered 1mM NaCl solution. A laser of wavelength 633 nm was used as source and detector was placed at 173° of angle. Measurements were carried-out at 25 °C. Zeta potential was determined by measuring electrophoretic mobility in a 0.22 µm-filtered 1 mM NaCl solution, using a Zetasizer NanoZSP from Malvern-Panalytical and a disposable folded capillary cell. A laser of wavelength 633 nm was used as source and detector was placed at 13° of angle. Measurements were carried-out at 25 °C. *A.* The hydrodynamic diameter (average size (Z-average) and polydispersity index (PdI) based on DLS), Zeta Potential (mV), drug loading (DL%) and encapsulation efficiency (EE%) of PAH-loaded i-Particles. Mean and SD of 4 measurements. *B.* Variation of hydrodynamic diameter (Z-average based on DLS, filled square and polydispersity index, PdI, empty squares) of iP-PAH (Drug Loading 8.49 %) with storage time (+ 4 °C, fridge). Four measurements were done and the averages and standard deviations are presented.

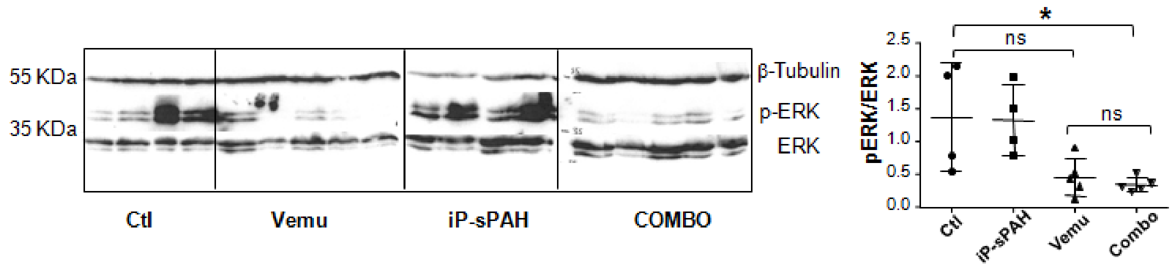

Figure S6. The addition of iP-sPAH to vemurafenib more strongly inhibits ERK phosphorylation in tumors. Tumor extracts were analyzed by western blotting with anti-Phospho-Erk1/2 and anti-Erk1/2 antibodies. Signal quantification was performed using ImageJ software and the pERK/ERK ratio was calculated and reported for each tumor extract. Significance is attained at P < 0.05 (*), ns: no significant difference.

A

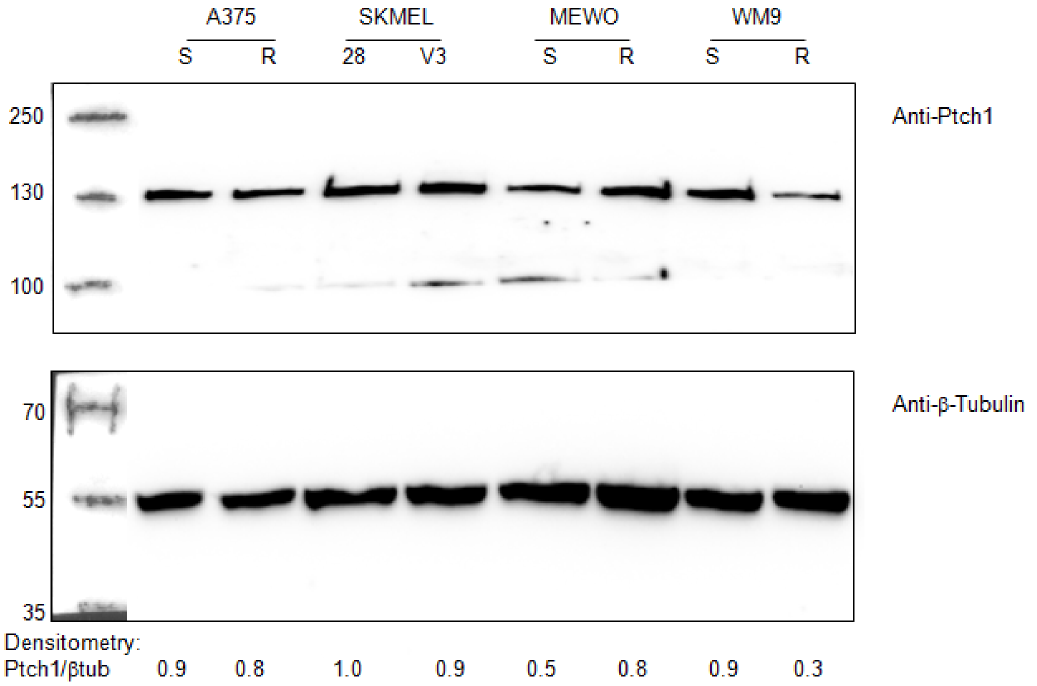

B

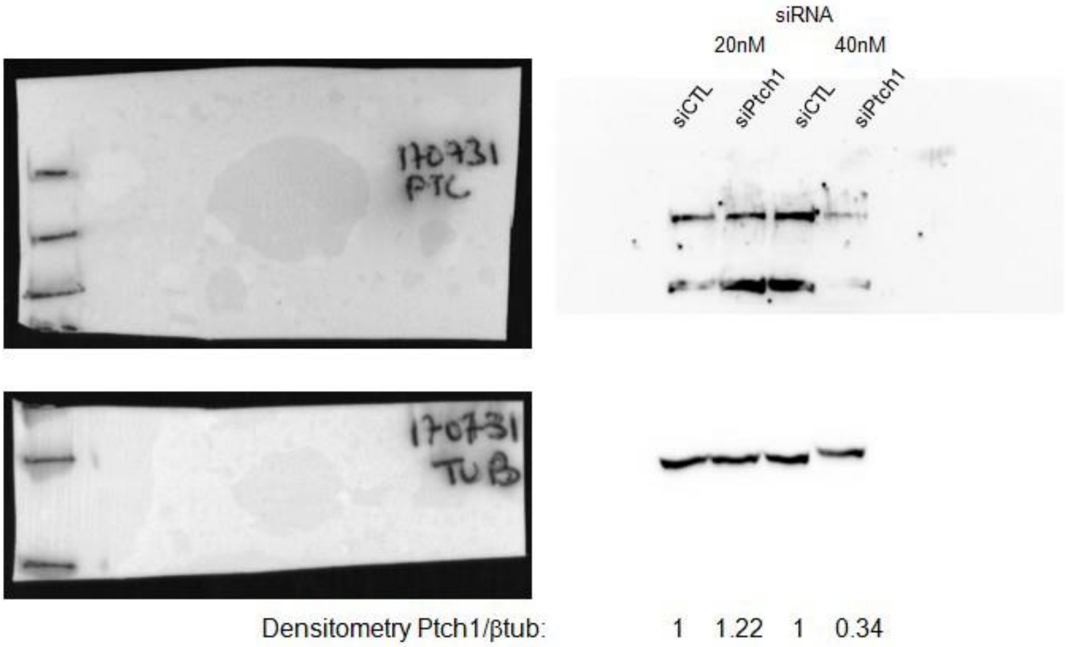
siRNA
20nM
40nM
siCTL
siPtch1
siCTL
siPtch1
170731 PTC
170731 TUB
Densitometry Ptch1/βtub:
1
1.22
1
0.34

C

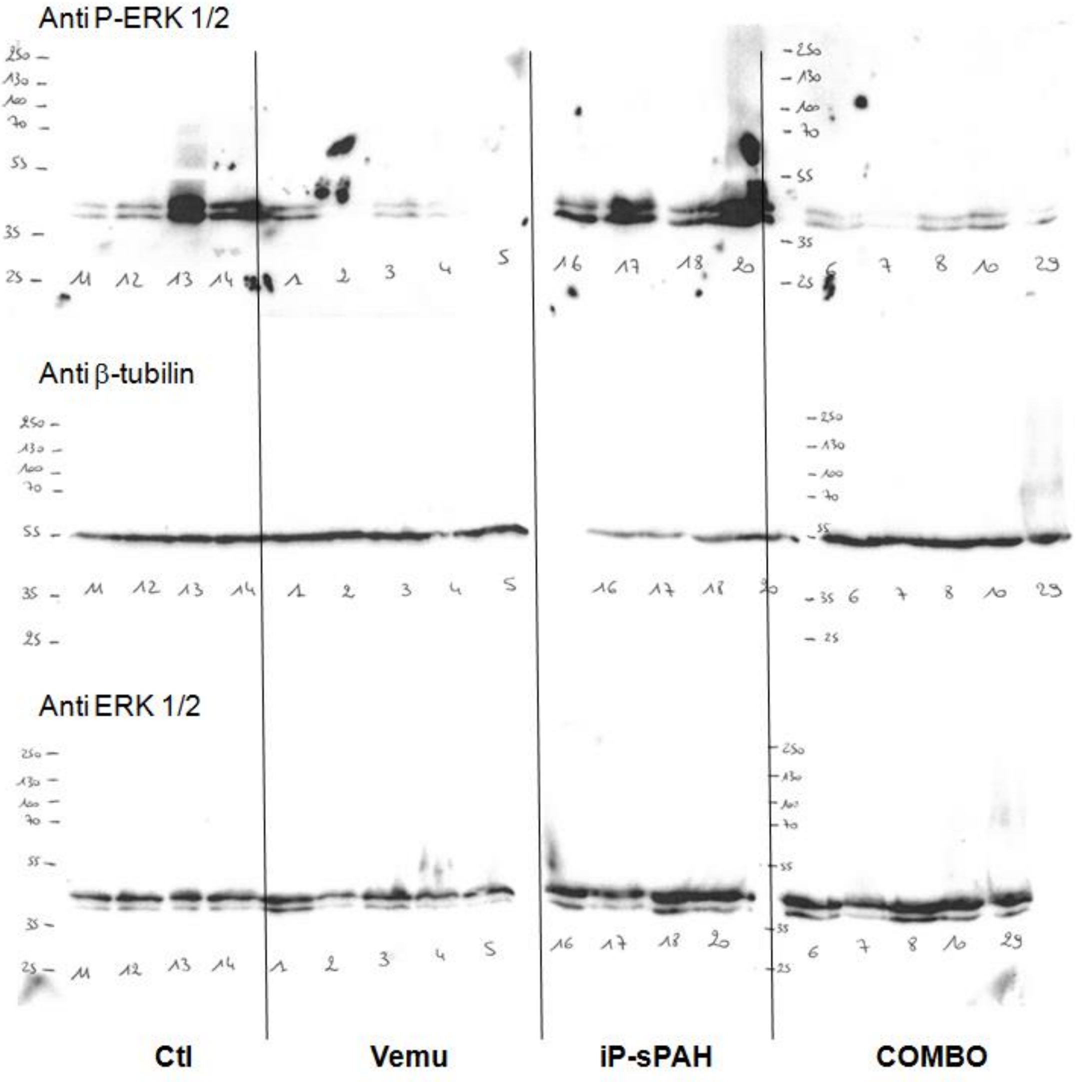
Anti P-ERK 1/2
Anti β-tubilin
Anti ERK 1/2
Ctl
Vemu
iP-sPAH
COMBO

Group	Tumor n°	Mouse id	Tubulin signal (AU)	ERK 1/2 signal (AU)	pERK1/2 signal (AU)	Ratio ERK/Tubulin	Ratio pERK/Tubulin	Ratio pERK/ERK
CTRL	11	1G1	30512	22365	12368	0,73	0,41	0,55
	12	1G3	44620	28695	22472	0,64	0,50	0,78
	13	1D5	48001	29997	64822	0,62	1,35	2,16
	14	2D6	49071	26434	53256	0,54	1,09	2,01
PAH	16	1D2	20592	42104	43212	2,04	2,10	1,03
	17	1G5	19885	32183	63990	1,62	3,22	1,99
	18	1D7	30559	52662	40882	1,72	1,34	0,78
	20	2D8	39644	42994	64778	1,08	1,63	1,51
Vemu	1	2D4	49443	31658	28596	0,64	0,58	0,90
	2	1D6	51127	16888	9005	0,33	0,18	0,53
	3	1D11	50443	28027	12326	0,56	0,24	0,44
	4	1G12	36198	23423	7511	0,65	0,21	0,32
	5	R12	45641	20235	2541	0,44	0,06	0,13
Combo	6	1G6	54167	45644	16728	0,84	0,31	0,37
	7	2D7	53674	30146	7056	0,56	0,13	0,23
	8	1G8	54728	51234	15783	0,94	0,29	0,31
	10	1G9	54557	44135	23446	0,81	0,43	0,53
	29	2D12	49038	36971	10807	0,75	0,22	0,29

Bonferroni's multip	Mean Diff,	95% CI of diff,	Significant?	Summary
CTRL vs. PAH	0,0475	-1,005 to 1,100	No	ns
CTRL vs. VEMU	0,911	-0,08758 to 1,910	No	ns
CTRL vs. COMBO	1,029	0,03042 to 2,028	Yes	*
PAH vs. VEMU	0,8635	-0,1351 to 1,862	No	ns
PAH vs. COMBO	0,9815	-0,01708 to 1,980	No	ns
VEMU vs. COMBO	0,118	-0,8235 to 1,059	No	ns

Figure S7. (**A**) Whole Western blot of Figure 1B. The membrane was cut into 2 pieces incubated respectively with anti-Ptch1antibody and anti-b tubulin antibody. (**B**) Whole Western blot of Figure 1C. V The membrane was cut into 2 pieces incubated respectively with anti-Ptch1antibody and anti-b tubulin antibody. (**C**) Whole Western blot of Supplementary Figure 6 and densitometry of each band. The membrane was incubated first with anti-phosphoERK antibody, then stripped and incubated with ERK antibody and then with anti-btubulin antibody. Densitometry of each band is shown in the table.
